# Evaluation of the Anti-Diabetic Activity of Some Common Herbs and Spices: Providing New Insights with Inverse Virtual Screening

**DOI:** 10.3390/molecules24224030

**Published:** 2019-11-07

**Authors:** Andreia S.P. Pereira, Antonio J. Banegas-Luna, Jorge Peña-García, Horacio Pérez-Sánchez, Zeno Apostolides

**Affiliations:** 1 Department of Biochemistry, Genetics and Microbiology, University of Pretoria, Pretoria Hillcrest 0083, South Africa; aspdpereira@gmail.com; 2 Structural Bioinformatics and High-Performance Computing Research Group (BIO-HPC), Universidad Católica de Murcia, 30107 Murcia, Spain; ajbanegas@alu.ucam.edu (A.J.B.-L.);

**Keywords:** herbs, spices, anti-diabetic, DIA-DB, virtual screening, sesquiterpenoids, flavonoids

## Abstract

Culinary herbs and spices are widely used as a traditional medicine in the treatment of diabetes and its complications, and there are several scientific studies in the literature supporting the use of these medicinal plants. However, there is often a lack of knowledge on the bioactive compounds of these herbs and spices and their mechanisms of action. The aim of this study was to use inverse virtual screening to provide insights into the bioactive compounds of common herbs and spices, and their potential molecular mechanisms of action in the treatment of diabetes. In this study, a library of over 2300 compounds derived from 30 common herbs and spices were screened in silico with the DIA-DB web server against 18 known diabetes drug targets. Over 900 compounds from the herbs and spices library were observed to have potential anti-diabetic activity and liquorice, hops, fennel, rosemary, and fenugreek were observed to be particularly enriched with potential anti-diabetic compounds. A large percentage of the compounds were observed to be potential polypharmacological agents regulating three or more anti-diabetic drug targets and included compounds such as achillin B from yarrow, asparasaponin I from fenugreek, bisdemethoxycurcumin from turmeric, carlinoside from lemongrass, cinnamtannin B1 from cinnamon, crocin from saffron and glabridin from liquorice. The major targets identified for the herbs and spices compounds were dipeptidyl peptidase-4 (DPP4), intestinal maltase-glucoamylase (MGAM), liver receptor homolog-1 (NR5A2), pancreatic alpha-amylase (AM2A), peroxisome proliferator-activated receptor alpha (PPARA), protein tyrosine phosphatase non-receptor type 9 (PTPN9), and retinol binding protein-4 (RBP4) with over 250 compounds observed to be potential inhibitors of these particular protein targets. Only bay leaves, liquorice and thyme were found to contain compounds that could potentially regulate all 18 protein targets followed by black pepper, cumin, dill, hops and marjoram with 17 protein targets. In most cases more than one compound within a given plant could potentially regulate a particular protein target. It was observed that through this multi-compound-multi target regulation of these specific protein targets that the major anti-diabetic effects of reduced hyperglycemia and hyperlipidemia of the herbs and spices could be explained. The results of this study, taken together with the known scientific literature, indicated that the anti-diabetic potential of common culinary herbs and spices was the result of the collective action of more than one bioactive compound regulating and restoring several dysregulated and interconnected diabetic biological processes.

## 1. Introduction

Diabetes is a chronic disease characterized by an insulin deficiency and/or insulin insensitivity, and was the seventh leading cause of death in 2016 [1,2,3,4]. It is a multi-organ disease affecting the pancreas, liver, muscles, kidney, and central nervous system and several complications such as hypertension, stroke, blindness, and kidney disease are associated with diabetes [5,6]. The main type of treatment for diabetes and controlling the associated hyperglycemia is in the form of insulin that primarily focuses on lowering and maintaining blood glucose levels [5]. However, insulin treatment is rather expensive and a somewhat invasive treatment strategy. In more recent years, since diabetes is a multifaceted disease, there has been an increase in the development of specific protein-targeted drugs, and specific inhibitors for targets like alpha-glucosidase, dipeptidyl peptidase-4 (DPP4), glucagon-like peptide-1 (GLP-1) receptor, and sodium-glucose co-transporter-2 (SGLT2) have been approved [6]. Unfortunately, some of these approved drugs have been met with several adverse effects [6]. As a better understanding of the pathogenesis and complexity in treating the disease arises, so too must the need for the development of more effective and safer drugs to treat the disease.

There is widespread traditional use in several cultures of decoctions prepared from medicinal plants in the treatment of diabetes [7,8,9,10,11]. In Chinese medicine, the belief is to use a more holistic approach that not only focuses on the treatment of the associated hyperglycemia but also on the associated diabetic complications [11]. Various reviews on medicinal plants effective in the treatment of diabetes can be found in the literature. Li et al 2004 reviewed 82 natural plant medicines used in Chinese traditional medicine for treating diabetes and included *Radix puerariae*, *Radix ginseng*, *Rhizoma anemarrhenae*, a mixture of the fruits, leaves and root epidermis of *Morus alba*, a mixture of *Radix paeoniae* and *Radix paeoniae alba*, *Allii sativi bulbus,* and *Gymnema sylvestre* [11]. These plants were found to contain more than one bioactive compound that besides improving blood glucose levels also improved the associated hyperlipidemia, improved insulin secretion, exerted antioxidant effects, improved renal function, and also treated diabetic retinopathy and neuropathy. Harlev et al. (2013) reviewed 22 desert and semi-desert plants commonly used in Bedouin ethnic medicine for the treatment of diabetes and included *Artemisia herba-alba*, *Teucrium polium*, *Ziziphus spina-christi*, *Larrea tridentate,* and *Balanites aegyptica* [12]. Compounds such as apigenin, cirsimaritin, christinin-A, nordihydroguaiaretic acid, isorhamnetin, and isorhamnetin-3-*O*-rutinoside were identified from these plants as having anti-diabetic properties. A review by Moradi et al. (2018) lists various medicinal plants found throughout the world that are effective in the treatment of diabetes and includes *Trigonella foenum-graecum* (India), *Ferula assafoetida* (Iran and Afghanistan), *Bauhinia forficate* (Argentina, Brazil and Peru), *Combretum micranthum* (Africa), *Liriope spicata* (East Asia and China), *Symplocos coccinea* (Mexico), as well as *Coccinia indica, Allium sativum,* and *Aloe vera. Burm* that are found distributed worldwide [13]. The biochemical mechanisms for the anti-diabetic activity of these plants identified included the stimulation of insulin secretion from pancreatic B-cells, inhibition of intestinal glucose digestion, and absorption as well as the regulation of enzymes such as lipoprotein lipase, glucose-6-phosphatase, lactate dehydrogenase, and aldose reductase. 

Plant secondary metabolites such as the flavonoids, terpenoids, alkaloids and polysaccharides that are found widespread in medicinal plants have been extensively studied for their anti-diabetic activity [14,15,16,17]. The flavonoids like quercetin, myricetin, kaempferol, and genistein have been found to protect pancreatic B-cells from damage, stimulate insulin secretion from B-cells, promote glucose uptake by the peripheral tissues, inhibit alpha-glucosidase and alpha-amylase, as well as promote glycogenesis [14]. Flavonoids have also been shown to have beneficial effects against diabetic complications such as diabetes-related cardiovascular disease, diabetic neuropathy, and retinopathy. Similarly, the terpenoids oleanolic acid, corosolic acid, betulinic acid, glycyrrhetinic acid, and gymnemic acid; the alkaloids berberine, catharanthine, vindoline, cryptolepine and trigonelline as well as polysaccharides isolated from tea, mulberry, ginseng, pumpkin, peach-gum, and guava have shown a diverse range of anti-diabetic effects in vitro and in vivo [15,16,17].

Herbs and spices are widely used in our daily lives as important seasonings and flavorings for our food. They are also commonly used for their health benefit properties such as antioxidant, anti-inflammatory, anticancer, anti-diabetic, antimicrobial, neuroprotective, and cardiovascular effects [18,19,20,21,22,23]. They represent attractive therapeutics interventions as they are complex mixtures of diverse compounds that can potentially and cooperatively modulate the activity of several dysregulated and interconnected disease targets. They are also widely available and are fairly inexpensive, with the exception of perhaps saffron. Although several studies can be found on the anti-diabetic activity of some of herbs and spices and in certain cases extensive scientific evaluations have been conducted, for the majority however, there is still a lack of scientific knowledge. The aim of this study was to provide insights into the bioactive compounds of these plants, as well as their molecular anti-diabetic mechanisms of action. 

In silico virtual screening methodologies are ideal for exploratory evaluations of the potential anti-diabetic activity of medicinal plants. As plants are complex mixtures of several different compounds, with in silico virtual screening methods, hundreds of compounds can be screened against multiple diabetes targets rapidly and cost effectively. This strategy has been employed to identify anti-cancer, anti-stroke, and anti-Alzheimer’s compounds from traditional Chinese medicines as well as their potential mechanisms of action [24,25,26]. In this study, we have implemented similar in silico methodologies to evaluate the anti-diabetic activity of 30 common herbs and spices.

## 2. Results and Discussion 

### 2.1. Literature Review

The anti-diabetic activity of some common herbs and spices were evaluated in this study with inverse virtual screening against 18 anti-diabetic drug targets with the DIA-DB web server. The aim of this study was to identify the bioactive compounds of these plants and provide insights into their molecular anti-diabetic mechanisms. Several studies can be found on the anti-diabetic activity of some of these herbs and spices, and the significant studies are summarized in Table 1 for in vivo and in vitro studies.

The primary in vivo model identified for studying the anti-diabetic activity of these plant extracts was either streptozotocin-induced or alloxan-induced diabetic rats. Aniseed [32], bay leaves [44,45,210], cardamom [211], cinnamon [66,211], cumin [212,213], dill [214], ginger [211], hops [118], rosemary [215], saffron [211,216], sage [217,218], and turmeric [219] have also been evaluated in type 2 diabetic patients. The major in vivo effects observed for the herbs and spices are a reduction in hyperglycemia and hyperlipidemia. The hyperglycemia observed in diabetes is the result of pancreatic dysfunction and insulin resistance, and is associated with unbalanced rates of glycogenolysis and gluconeogenesis resulting in increased endogenous glucose production [220]. The reduction in hyperlipidemia was observed as decreases in total cholesterol, low-density lipoprotein (LDL), very-low-density lipoprotein (VLDL) and triglyceride levels with an increase in the high-density lipoprotein (HDL) levels. Diabetes is characterized by low plasma HDL and high triglycerides, cholesterol, and LDL levels [221]. Increased levels of LDL inhibit insulin secretion and induce pancreatic B-cell apoptosis, while an increase in HDL protects against apoptosis and improves B-cell function, reduces plasma glucose and increases plasma insulin. An accumulation of triglycerides in the liver, pancreas, and muscles is correlated with insulin resistance and the cholesterol levels in adipocytes increase with increasing levels of triglycerides [222]. 

With the effect on serum insulin levels, the type of model used namely type 1 or type 2 diabetic model plays a role. This is particularly seen with cumin, liquorice, and marjoram that can increase or decrease serum insulin levels depending on the insulin status of the control diabetic rat model. Type 1 diabetes is associated with an insulin deficiency while in type 2 diabetes normal insulin or raised insulin levels are observed with significant insulin resistance [1,2,3]. Type 2 diabetes, however, may eventually progress to an insulin deficient state following pancreatic B-cell dysfunction as a result of exhaustive insulin production and secretion [2]. The herbs and spices observed to raise the serum insulin levels and have possible benefits in the treatment of insulin-deficient diabetes were black pepper, caraway, fennel, fenugreek, ginger, lemon balm, oregano, parsley, rosemary, saffron, sage, turmeric, and yarrow. Cardamom, cinnamon, dill, lemongrass, and nutmeg were found to decrease serum insulin levels and may be beneficial in the treatment of hyperinsulinemia type diabetes. 

Some detailed studies exploring the in vivo anti-diabetic activity of some of the herbs and spices can be seen in Table 1. This is true for cinnamon, clove, fenugreek, hops, liquorice, marjoram, ginger, nutmeg, rosemary, and turmeric, where authors have evaluated the specific effects of these extracts on the expression and activity of some anti-diabetic drug targets like phosphoenolpyruvate carboxykinase (PEPCK), PPARA/G, glucose 6-phosphatase (G6Pase), glucose transporter type 4/2 (GLUT4/2), pyruvate kinase, alpha-amylase, alpha-glucosidase, SGLT1, hexokinase, lipoprotein lipase, fatty acid synthase, and fructose-1,6-bisphosphatase (FBP1). For the majority of the herbs and spices presented here, however, very little is known regarding their observed anti-diabetic activity. 

The major in vitro studies have focused on the inhibitory activity of the herbs and spices on alpha-glucosidase and alpha-amylase in particular yeast or rat intestinal alpha-glucosidase and porcine pancreatic alpha-amylase (Table 1). Alpha-amylase is responsible for the digestion of dietary starch to maltase that in turn is digested into glucose by intestinal alpha-glucosidase. Inhibition of these two enzymes will delay carbohydrate digestion thus lowering the postprandial blood glucose level [223]. Majority of the herbs and spices were found to inhibit these two enzymes regulating carbohydrate metabolism. However, varying levels of activity could be found for a single plant dependent on extract preparation and assay conditions, with some studies showing a high level of inhibitory activity, while no activity was observed in other studies. This has also been noted in a detailed review on the alpha-glucosidase and alpha-amylase inhibitory activity of several medicinal plants [68]. The inhibitory activity of some herbs and spices on other diabetic targets such as pancreatic lipase and aldose reductase as well as transactivation of the PPARs was also observed.

For some herbs and spices, individual bioactive compounds have also been identified and include piperine for black pepper [50,54], cinnamaldehyde and cinnamatannin B1 for cinnamon [66], dehydrodieugenol, dehydrodieugenol B, oleanolic acid and maslinic acid for clove [74,77], cuminaldehyde for cumin [82], diosmin, [6]-gingerol, carvacrol and thymol for ginger [107,112], 3′-geranylchalconaringenin, xanthohumol, isohumulone and isocohumulone for hops [114,117,118], citronellol for lemongrass [127], glycyrrhizin, 18b-glycyrrhetinic acid, liquiritigenin, isoliquiritigenin, glabridin and licochlacone A for liquorice [131,133,134,135,137,138,143], 6-hydroxyapigenin for marjoram [145], macelignan, licarin B, tetrahydrofuroguaiacin B, nectandrin B, nectandrin A and dihydroguaiaretic acid for nutmeg [149,151,153,154], rosmarinic acid and salvianolic acid B for oregano [159], capsaicin for paprika [164], rosmarinic acid, carnosol, carnosic acid, luteolin, 7-*O*-methylrosmanol, hispidulin, and cirsimaritin for rosemary [144], safranal and crocin for saffron [180], and ar-turmerone, curcumin, demethoxycurcumin, bisdemethoxycurcumin, and tumerin for turmeric [194,195,198]. Considering that herbs and spices are complex mixtures of diverse compounds, it is likely that the anti-diabetic activity of a given plant is dependent on multiple compounds regulating the activity of several anti-diabetic drug targets. This can be seen for clove, ginger, hops, liquorice, nutmeg, rosemary, and turmeric, where more than one compound has been identified as being the potential bioactive compound responsible for the observed anti-diabetic effects of these plants. 

### 2.2. Inverse Virtual Screening with the DIA-DB Web Server

A compound library of over 2300 compounds from literature was generated for the herbs and spices presented in this study and the compounds were subsequently screened with the DIA-DB webserver (http://bio-hpc.eu/software/dia-db/). The DIA-DB webserver employs inverse virtual screening of compounds with Autodock Vina against a given set of 18 protein targets associated with diabetes [224]. These targets were aldose reductase (AKR1B1), AMY2A, DPP4, FBP1, free fatty acid receptor 1 (FFAR1), glucokinase (GCK), 11B-hydroxysteroid dehydrogenase type 1 (HSD11B1), insulin receptor (INSR), MGAM, NR5A2, pyruvate dehydrogenase kinase isoform 2 (PDK2), PPARA, PPARD, PPARG, PTPN9, liver glycogen phosphorylase (PYGL), RBP4, and retinoid X receptor alpha (RXRA). The major function of each of the protein targets can be found in Table 2 and have been broadly divided into three categories representing either their mode of action on insulin secretion and/or sensitivity, regulation of glucose metabolism, or regulation of lipid metabolism. Although these protein targets have been broadly divided into three major categories, their activity can be interconnected with one or more protein targets found in a different category. This is observed from evidence in literature on the regulation of GCK by NR5A2 and PPARG [225], GCK can act as a glucose sensor in pancreatic B-cells and stimulate insulin secretion [226], regulation of PDK2 activity by PPARA and PPARD [227], FFAR1 regulation by PPARG [228], HSD11B1 regulation by PPARG [229], and PPARG can promote insulin sensitivity by enhancing the expression and translocation of the GLUT transporters responsible for glucose uptake [229]. 

A cutoff docking score for each of the protein targets was set to distinguish potentially active compounds from the inactive ones. The cutoff docking score was decided on by the average docking score obtained for known and/or experimental drugs together with the crystallized ligand found in the active site of a given protein target (Table S1). The cutoff docking scores set for each of the protein targets can be found in Table 2 as well as the total number of compounds identified as potential inhibitors for each of the targets. Of the library compounds submitted to the DIA-DB web server over 940 compounds were identified as potential anti-diabetic bioactive compounds. The herbs and spices presented here were thus found to be very rich sources of anti-diabetic compounds. Over 200 potential agonists/inhibitors were identified for protein targets AMY2A, DPP4, FBP1, MGAM, NR5A2, PPARA, and RBP4. 

A summary of the inverse virtual screening results can be found in Table 3. Table S2 contains the full list of bioactive compounds identified and their source plants. As can be seen for majority of the herbs and spices, the number of compounds found to be potentially responsible for the observed anti-diabetic activity accounted for 30%–50% of the total number of compounds submitted. More than half of the compounds submitted for liquorice, hops, fennel, and rosemary were found to be potential bioactive compounds with 73%, 61%, 54%, and 54%, respectively. This is also represented in the literature, where a large volume of detailed studies on the anti-diabetic activity of these plants can be found. Only a few (9%) of the 166 compounds submitted for paprika were found to be potential bioactive compounds. This is also reflected in the literature on the anti-diabetic activity of paprika (*Capsicum annuum*), where only a few studies were found and the activity noted was somewhat moderate. Surprisingly, allspice and yarrow where only a few studies on their anti-diabetic activity can be found, had a high percentage 49% of potential bioactive compounds that were identified. 

A relative low number of bioactive compounds were identified for ginger (25%). This was unexpected as several detailed studies can be found for the anti-diabetic activity of this particular plant. This indicates that the targets identified here can only partly explain the observed anti-diabetic activity of ginger and that the effect of ginger on other anti-diabetic targets such as pancreatic lipase, GLUT4, phosphofructokinase, and pyruvate kinase also account for the observed reduction in hyperglycemia and hyperlipidemia. This is true for all the herbs and spices presented in this study, as diabetes as a disease is a complex one involving multiple dysregulated processes across several organ systems like the liver, muscles, adipose tissue, and pancreas [5,230]. This indicates that the regulation of various protein targets is needed and that the effect on protein targets other than those presented here may also play a role. This study, however, has provided new insights into the anti-diabetic activity of ginger as well as identified new potential protein targets for the bioactive compounds of ginger. Also, of note is that the bioactive compounds identified in this study were the gingerols and shogaols that have been identified in the literature as the major bioactive compounds of ginger.

Since diabetes is such a complex disease process, the need arises for multi-targeted compounds rather than a “single target–single drug approach” [242,243,244]. This is also why plants such as the herbs and spices presented here are attractive treatments for diabetes as multiple protein targets can be regulated with more than one compound. In this study nearly half of the herbs and spices were found to contain a large percentage of multi-protein-targeted compounds and included cinnamon, cumin, fennel, fenugreek, lemon balm, lemongrass, liquorice, marjoram, oregano, rosemary, saffron, sage, ad thyme. 

**Figure 1:**
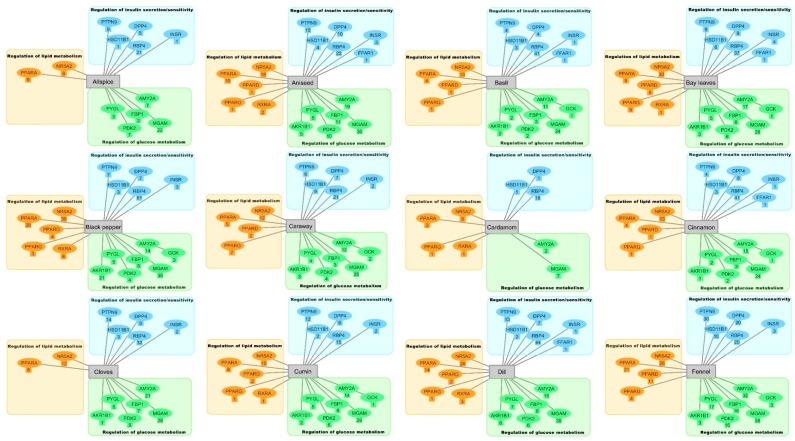

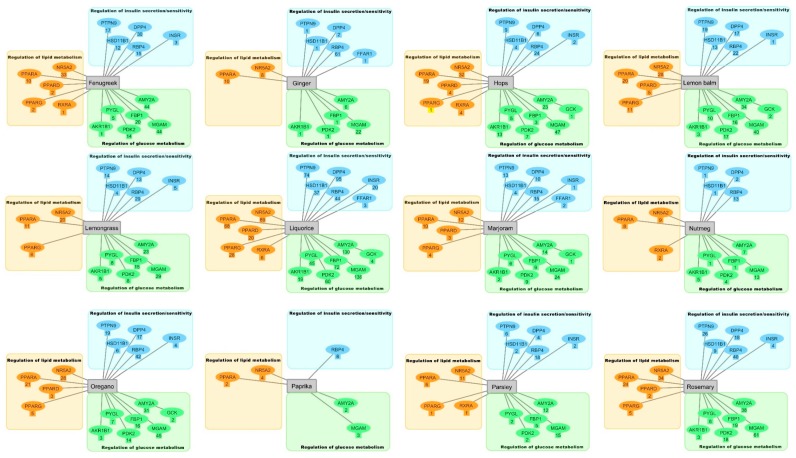

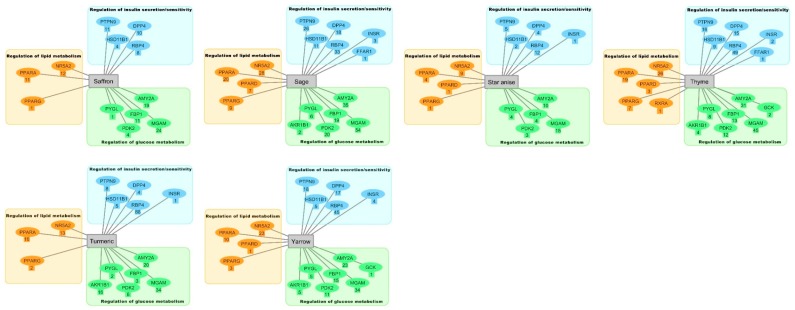
Protein-compound target networks identified for each herb and spice. The number below each protein target denotes the number of potential bioactive compounds identified.

The major anti-diabetic effects observed in the literature were a reduction in hyperglycemia, reduction in hyperlipidemia, and regulation of insulin secretion. In Figure 1, the effects of the individual herbs and spices on these three diabetes hallmarks dependent on the protein targets of the DIA-DB webserver can be seen. All the herbs and spices were found to be potential regulators of 12 or more of the protein targets with the exception of paprika and cardamom, whose compounds were only found to be potential regulators of 5 and 9 targets, respectively. The reduction in hyperglycemia can be attributed to regulation of the protein targets involved in glucose metabolism. Inhibition of AMY2A and MGAM will delay carbohydrate digestion, thus lowering the postprandial blood glucose level [223]. Inhibition of FBP1 and PYGL will inhibit endogenous glucose production by the liver through the inhibition of gluconeogenesis and glycogenolysis, respectively, thereby reducing blood glucose levels [220]. The PDKs are upregulated in diabetes and are responsible for inhibiting the pyruvate dehydrogenase kinase complex that in turn is responsible for the conversion of pyruvate into acetyl-CoA that then enters the Krebs cycle [245]. By inhibiting PDK2, the serum glucose levels can be reduced through the inhibition of pyruvate availability for liver gluconeogenesis [246]. Activation of GCK will also lead to a reduction in serum glucose levels by promoting glycogenesis and glycolysis through the phosphorylation of glucose to glucose-6-phosphate [226]. 

The reduction in the observed hyperlipidemia can be attributed to regulation of the protein targets NR5A2, the PPARs and RXRA that are involved in lipid metabolism. The PPARs play various roles in lipid metabolism by regulating the genes involved in lipogenesis, triglyceride synthesis, reverse cholesterol transport, lipolysis, and fatty acid oxidation. Stimulation of PPARG induces the expression of cluster of differentiation 36 (CD36) that promotes the removal of oxidized LDL from the blood by the macrophages [247]. PPARG also induces the expression of the liver X receptor (LXR) that in turn induces the expression of the reverse cholesterol transporter ABCA1 which releases HDL into the bloodstream, where the cholesterol is converted to bile salts in the liver and is subsequently excreted. PPARG also promotes adipogenesis forming new adipocytes that are able to take up excess lipids from the plasma while promoting apoptosis of lipid-saturated adipocytes [229]. PPARA in the liver promotes fatty acid oxidation, increases fatty acid uptake by increasing the expression of fatty acid transport protein and fatty acid translocase, increases apolipoprotein A-1 (ApoA-1, component of HDL), decreases ApoC-2 (component of VLDL), and increases lipoprotein lipase (promotes breakdown of triglycerides into fatty acids) [229].

PPARD like PPARA promotes fatty acid oxidation through the upregulation of target gene carnitine palmitoyltransferase A1 and decreases triglyceride levels through the downregulation of the target protein angiopoietin-like 4 protein that is responsible for inhibiting the breakdown and clearance of triglycerides [229,241]. Treatment with PPAR agonists will thus result in decreased cholesterol, triglyceride, LDL, and VLDL levels, while increasing HDL levels. NR5A2 is highly expressed in the liver and its targets are the bile-acid synthesizing enzymes cholesterol 7-alpha hydroxylase (CYP7A1) and sterol 12-alpha hydroxylase (CYP8B1). Other targets genes include mediators of cholesterol uptake and efflux, HDL formation, cholesterol exchange between lipoproteins, and fatty acid synthesis [225]. Treatment with agonists of NR5A2 would thus also result in reduced hyperlipidemia. 

The targeting of proteins PTPN9, DPP4, HSD11B1, RBP4, FFAR1, and INSR will promote insulin secretion from the B-cells and improve insulin sensitivity. This in turn will also promote glucose homeostasis and reduce hyperglycemia. PTPN9 disrupts the insulin signaling pathway and thus treatment with inhibitors will result in insulin sensitization and improve glucose homeostasis [248]. Inhibition of DPP4 will increase the half-life of the incretin hormones, thereby increasing insulin secretion and allowing time to normalize blood glucose levels [249]. Compounds capable of inhibiting HSD11B1 can inhibit glucose production by the liver and improve glucose-dependent insulin sensitivity [250]. Elevated levels of RBP4 are associated with insulin resistance where RBP4 acts as an adipokine disrupting insulin signaling and decrease glucose uptake in the muscles [251,252]. RBP4 also promotes glucose production by the liver thus increasing plasma glucose levels. Compounds that are thus able to bind RBP4 may prevent its association with transthyretin, resulting in enhanced clearance of the elevated serum RBP4 through the kidneys [252]. Treatment with FFAR1 agonists will stimulate glucose-dependent insulin secretion from the pancreatic B-cells and in the gastrointestinal tract will stimulate the release of the incretin hormones [253]. Activation of INSR by agonists will stimulate the insulin signaling pathway, thereby improving insulin sensitivity and promoting glucose uptake by the tissues [254]. 

Some individual bioactive compounds for the some of the herbs and spices have been identified in previous studies. However, the results presented here indicate that the anti-diabetic effects of these plants rather arise from several compounds regulating multiple protein targets whose biological roles are interconnected. Extracts prepared from these herbs and spices can thus comprehensively treat the multiple dysregulated processes associated with diabetes. The results presented in Figure 1 provide insights into the anti-diabetic activity of allspice, aniseed, basil, bay leaves, black pepper, caraway, cardamom, dill, fennel, lemongrass, parsley, saffron, sage, star anise, thyme, and yarrow, where anti-diabetic activity has been identified but studies evaluating their anti-diabetic mechanisms of action are lacking. For herbs and spices like cinnamon, clove, cumin, fenugreek, ginger, liquorice, marjoram, nutmeg, oregano, rosemary, and turmeric, the results present here provide new insights, build-on and support their well-established anti-diabetic activity. For example, in vivo studies have found that treatment with rosemary modulates the activity of GCK and FBP1. Although no agonists for GCK were identified by the DIA-DB webserver, agonists for NR5A2 and PPARG that can regulate the activity of GCK were identified [225]. This was also observed for fenugreek where 33 NR5A2 and 2 PPARG agonists were identified. For FBP1 inhibition by rosemary, 19 compounds were identified as potential inhibitors and include several flavonoid glucosides like 6-hydroxyluteolin-7-*O*-glucoside, Apigenin-7-*O*-glucoside, hispidulin-rutinoside, hesperidin and luteolin-7-*O*-glucoside, luteolin-7-*O* glucuronide and luteolin-7-*O*-rutinoside. Treatment with rosemary has also been associated with in vitro and in vivo alpha-glucosidase inhibitory activity and this study is in agreement as 61 compounds were identified as possible inhibitors of MGAM. Hops compounds have been found to modulate the expression of several proteins involved in lipogenesis, triglyceride synthesis, reverse cholesterol transport, lipolysis, and fatty acid oxidation. The three PPARs have been shown to modulate the expression of these target proteins and in this study 19 PPARA, 4 PPARD, and 1 PPARG agonists were found and were predominantly the geranyl- and prenyl-tetrahydroxychalcones and the xanthohumols. Similarly, and in agreement with the literature, for fenugreek, PPARA, and PPARG agonists were also identified, as were PYGL, AMY2A, and MGAM inhibitors. 

### 2.3. Hierarchical Clustering Analysis

Hierarchical clustering analysis of the bioactive compounds identified in each herb and spice was performed using Tanimoto similarities to determine whether the bioactive compounds identified showed some chemical similarity in structure [24,25,26]. The results of the clustering analysis are shown in Table 4. No clustering was found for caraway, cardamom, cinnamon, marjoram, nutmeg, oregano, and paprika. The number of chemically similar compounds within these herbs and spices may be inadequate to create meaningful clusters. The two major chemical classes identified in the herbs and spices were the sesquiterpenoids and the flavonoids/flavonoid glycosides. The sesquiterpenoids are one of the major types of compounds that can be found in the volatile oils of plant extracts and have been found to have anti-diabetic activity [255]. The volatile oils of basil, bay leaves, black pepper, clove, lemongrass, and turmeric were found to have anti-diabetic activity in vitro and/or in vivo [35,40,43,48,70,129,130,202]. The flavonoids and flavonoid glycosides were a major representative chemical class of the bioactive compounds found in aniseed, bay leaves, clove, cumin, dill, fennel, fenugreek, lemon balm, lemongrass, liquorice, parsley, rosemary, saffron, sage, thyme, and yarrow. Several studies can be found on the anti-diabetic activity of flavonoids and their glycosides [14,256,257,258,259,260]. 

## 3. Materials and Methods

### 3.1. Literature Review

The literature review on the anti-diabetic activity of the herbs and spices was conducted with Google Scholar [261] and ScienceDirect [262] using the following search terms: “common plant name” such as fenugreek, liquorice or sage, and so forth or “scientific plant name” such as *Trigonella foenum-graecum*, *Glycyrrhiza glabra* or *Salvia officinalis*, and so forth, together with “anti-diabetic” or “diabetes” or “individual protein target” such as alpha-glucosidase, alpha-amylase or PPAR, and so forth. To build the compound library for virtual screening, the search terms of “common plant name” or “scientific plant name” together with “bioactive compounds”, “liquid chromatography”, “mass-spectrometry”, “gas chromatography”, “phenolic compounds” or “essential oil”, were used. The FooDB was also consulted [263].

### 3.2. Preparation of Compound Structures and Inverse Virtual Screening of Potential Anti-Diabetic Activity.

The SMILES notations of the compounds were obtained directly from PubChem [264]. When the compound was not found in PubChem the two-dimensional structure of the compounds was created with Advanced Chemistry Development (ACD)/ChemSketch freeware version 12.02, (Advanced Chemistry Development, Inc., Toronto, ON, Canada) [265] and then converted to its representative SMILES notation.

The SMILES notation of each compound was subsequently submitted to the DIA-DB webserver that employs inverse virtual screening of compounds with Autodock Vina against a given set of 18 protein targets associated with diabetes [224]. These targets were AKR1B1, DPP4, FBP1, FFAR1, GCK, HSD11B1, INSR, MGAM, PYGL, NR5A2, AMY2A, PPARA, PPARD, PPARG, PTPN9, PDK2, RXRA, and RBP4. 

A cutoff docking score based on the average docking score of a set of known and/or experimental inhibitors was set to distinguish between potential active and inactive compounds (Table S1). The predicted compound-target network was generated by Cytoscape version 3.4.0 (Cytoscape Consortium, San Diego, CA, USA) [266] to explore the potential anti-diabetic mechanisms of action. 

### 3.3. Hierarchical Clustering Analysis of the Bioactive Compounds

Hierarchical clustering analysis was performed for the bioactive compounds identified from each herb and spice with Schrödinger Canvas Suite version 3.2.013 (Schrödinger, LLC, New York, NY, USA) [267]. The molecular fingerprint was calculated from the two-dimensional structure of the compounds in the form of extended connectivity fingerprint 4 (ECFP4). From these fingerprints, hierarchical clustering analysis was performed using the metric of Tanimoto similarity and the Average cluster linkage method that clusters according to the average distance between all inter-cluster pairs. 

## 4. Conclusions

The herbs and spices presented here were found to be rich sources of compounds with potential anti-diabetic activity through the use of the DIA-DB webserver. Over 900 compounds from the herb and spices library were found to have potential anti-diabetic activity and the two major chemical classes of bioactive compounds observed were the sesquiterpenoids and the flavonoids/ flavonoid glycosides. For majority of the herbs and spices, between 15 (paprika) and 157 (liquorice) compounds were identified as potential bioactive compounds versus the literature where the focus had been on only one to six compounds for a given herb or spice. The major anti-diabetic effects found in the literature for the herbs and spices were a reduction in hyperglycemia, reduction in hyperlipidemia, and regulation of insulin secretion and while some detailed studies exploring the anti-diabetic mechanisms of action of some of the herbs and spices could be found, for the majority presented here, however, very little was known. We found that the biological functions of the DIA-DB diabetes drug targets to be associated with glucose and lipid homeostasis as well as insulin secretion and sensitivity and that some of these targets were interconnected. Thus, through the regulation of these targets by the herb and spices compounds, we could explain the observed in vivo anti-diabetic activity of the herbs and spices. Collectively, the compounds of a particular herb or spice were observed to be potential regulators of 12 or more of the DIA-DB diabetes drug targets and in several cases more than one compound could potentially regulate a particular protein target, while in some cases one compound could potentially regulate more than one protein target. Bay leaves, liquorice and thyme were found to contain compounds that could potentially regulate all 18 protein targets followed by black pepper, cumin, dill, hops and marjoram with 17 protein targets. It was found that through this multi-compound-multi-target regulation of these specific diabetes targets that the major in vivo anti-diabetic effects observed in literature for the herbs and spices could be explained. We provided new insights in to the anti-diabetic mechanisms of action of allspice, aniseed, basil, bay leaves, black pepper, caraway, cardamom, dill, fennel, lemongrass, parsley, saffron, sage, star anise, thyme, and yarrow that was poorly characterized, while supporting and building onto the established anti-diabetic mechanisms of cinnamon, clove, cumin, fenugreek, ginger, liquorice, marjoram, nutmeg, oregano, rosemary, and turmeric. Extracts prepared from these herbs and spices can thus comprehensively treat the multiple dysregulated and interconnected processes associated with diabetes.

## Figures and Tables

**Table 1:** Literature review on the in vitro and in vivo anti-diabetic activity of various herbs and spices.

Plant Name	Common Name	Part Evaluated	In Vitro Anti-Diabetic Effects	In Vivo Anti-Diabetic Effects	References
*Pimenta Dioica*	Allspice	Berries	Alpha-glucosidase and alpha-amylase inhibitory, increased insulin-stimulated glucose metabolism in adipocytes	Streptozotocin-induced diabetic rats-improves antioxidant status	[27,28,29]
*Pimpinella anisum*	Aniseed	Seeds	Alpha-glucosidase, alpha-amylase, HMGR and pancreatic lipase inhibitory activity	Diabetic patients-reduced hyperglycemia, reduced hyperlipidemia, improved antioxidant status	[30,31,32]
*Ocimum basillicum*	Basil	Leaves	Alpha-glucosidase, alpha-amylase, aldose reductase, pancreatic lipase inhibitory activity, increases insulin-stimulated glucose metabolism in adipocytes, increase GLUT4 translocation	Alloxan/streptozotocin-induced diabetic rats-reduced hyperglycemia, reduced hyperlipidemia, improved antioxidant status, increased liver glycogen content, improved liver function	[27,33,34,35,36,37,38,39,40,41,42]
*Laurus nobilis*	Bay leaves	Leaves	Alpha-glucosidase inhibitory activity; increases insulin-stimulated glucose metabolism in adipocytes	Type 2 diabetic patients-reduced hyperglycemia, reduced hyperlipidemia	[27,43,44,45]
*Piper nigrum*	Black pepper	Fruit and leaves	Alpha-glucosidase, alpha-amylase and aldose reductase inhibitory activity, increased glucose consumption by adipocytes, induced transactivation of PPARA	Alloxan/streptozotocin-induced diabetic rats-reduced hyperglycemia, reduced hyperlipidemia, increased serum insulin levels, improved antioxidant status, improved liver function	[40,42,46,47,48,49,50,51,52,53,54]
*Carum carvi*	Caraway	Fruit/seeds	Induced transactivation of PPARA	Alloxan/streptozotocin-induced diabetic rats-reduced hyperglycemia, reduced hyperlipidemia, increased serum insulin levels, improved antioxidant status	[49,55,56,57,58,59]
*Elettaria cardamomum*	Cardamom	Seeds and leaves	No significant studies identified	Alloxan-induced diabetic rats-reduced hyperglycemia, reduced hyperlipidemia, decreased plasma insulin levels, improved liver function	[60,61,62,63]
*Cinnamomum verum*	Cinnamon	Bark	Alpha-glucosidase, alpha-amylase, aldose reductase inhibitory activity, increased insulin-stimulated glucose metabolism in adipocytes, increased expression and translocation of GLUT4 and GLUT1, induced transactivation of PPARA and PPARG	Alloxan/streptozotocin-induced diabetic rats-reduced hyperglycemia, reduced hyperlipidemia, increased plasma insulin levels, improved liver function, increased GLP1 levels, increased pyruvate kinase activity, decreased PEPCK activity	[27,42,60,64,65,66,67,68,69]
*Syzygium aromaticum*	Clove	Flower buds	Alpha-glucosidase, alpha-amylase, PEPCK and G6Pase inhibitory activity, increased insulin-stimulated glucose metabolism in adipocytes, induced transactivation of PPARG	Streptozotocin-induced diabetic rats-reduced hyperglycemia, reduced hyperlipidemia, improved antioxidant status, improved liver function, reduced expression of GLUT2, SGLT1, alpha-amylase and alpha-glucosidase in rat small intestine, increased glycogen content of liver and muscles, increased activity of hexokinase in liver and muscle	[27,68,69,70,71,72,73,74,75,76,77,78,79]
*Cuminum cyminum*	Cumin	Seeds	Alpha-glucosidase, alpha-amylase, aldose reductase inhibitory activity, induced transactivation of PPARG, stimulated glucose uptake in myotubes	Streptozotocin-induced diabetic rats-reduced hyperglycemia, reduced hyperlipidemia, reduced/ increased serum insulin levels depending on model, improved antioxidant status, increased liver and skeletal muscle content	[42,70,80,81,82,83,84,85]
*Anethum graveolens*	Dill	Aerial parts and seeds	No significant studies identified	Alloxan/streptozotocin-induced diabetic rats-reduced hyperglycemia, reduced hyperlipidemia, improved antioxidant status	[86,87,88,89]
*Foeniculum vulgare*	Fennel	Seeds and leaves	Alpha-glucosidase, alpha-amylase, aldose reductase inhibitory activity, increased glucose consumption by adipocytes	Alloxan/streptozotocin-induced diabetic rats-reduced hyperglycemia, reduced hyperlipidemia, increased serum insulin levels, improved antioxidant status, improved liver function, increased liver glycogen content, increased liver and kidney hexokinase activity	[40,42,90,91,92,93,94,95]
*Trigonella foenum-graecum*	Fenugreek	Seeds	Alpha-glucosidase, alpha-amylase, aldose reductase, pancreatic lipase inhibitory activity, induced transactivation of PPARG, PPARD and PPARA	Alloxan/streptozotocin-induced diabetic rats-reduced hyperglycemia, reduced hyperlipidemia, increased serum insulin levels, improved antioxidant status, improved liver function, increased liver, muscle and kidney glycogen content, reduced activity of intestinal maltase, sucrase and lactase, intestinal lipase, alpha-amylase, glycogen phosphorylase and G6Pase, increased activity of glycogen synthase, hexokinase, PPARG, PPARA and glucose-6-phosphate dehydrogenase	[42,69,96,97,98,99,100,101,102,103,104,105,106]
*Zingiber officinale*	Ginger	Root	Alpha-glucosidase, alpha-amylase, aldose reductase, pancreatic lipase inhibitory activity, increased GLUT4, increased glucose consumption by adipose tissues	Alloxan/streptozotocin-induced diabetic rats-reduced hyperglycemia, reduced hyperlipidemia, increased serum insulin levels, improved liver function, increased activity of liver glucokinase, phosphofructokinase, and pyruvate kinase	[65,101,107,108,109,110,111,112]
*Humulus lupulus*	Hops	Cones and leaves	Alpha-glucosidase, alpha-amylase, aldose reductase, pancreatic lipase inhibitory activity, induced PPARG and PPARA transactivation; induced FXR activity	Streptozotocin-induced diabetic rats-reduced hyperglycemia, reduced hyperlipidemia, increased hepatic glycogen content, reduced expression of hepatic GLUT2 and hepatic acetyl-CoA carboxylase, increased hepatic FAS expression. Diabetic KK-Ay mice-reduced hyperglycemia, reduced hyperlipidemia, increased expression of acyl-CoA oxidase, fatty acid translocase, lipoprotein lipase and PPARA, reduced expression of SRE-BP1, FAS, AceCS, SCD-1, ACL, PEPCK, G6Pase, and FBP1.	[113,114,115,116,117,118,119,120]
*Melissa officinalis*	Lemon balm	Leaves	Alpha-glucosidase, alpha-amylase, pancreatic lipase inhibitory activity, induced activation of PPARA, PPARD, and PPARG, increased glucose consumption through adipocytes, increased expression of SREBP1, FABP4, fatty acid transport protein 4, CD36 molecule, PDK4, LXRA, lipogenic stearoyl CoA desaturase	Alloxan/streptozotocin-induced diabetic rats-reduced hyperglycemia, reduced hyperlipidemia, increased serum insulin levels	[40,101,121,122,123,124,125]
*Cymbopogon citratus*	Lemongrass	Leaves	Alpha-glucosidase, alpha-amylase, aldose reductase inhibitory activity	Poloxamer-47-induced type 2 diabetic rats-reduced hyperglycemia, reduced hyperlipidemia, reduced serum insulin levels and insulin resistance, improved antioxidant status, increased GLP1 expression	[126,127,128,129,130]
*Glycyrrhiza glabra*	Liquorice	Root	Alpha-glucosidase, alpha-amylase, aldose reductase, PTP1B inhibitory activity, induced PPARG activation, increased insulin-stimulated glucose uptake by adipocytes, stimulated glucose-mediated insulin secretion from pancreatic islet cells, increased the expression of PDX-1 and GCK	Streptozotocin-induced diabetic rats-reduced hyperglycemia, reduced hyperlipidemia, increased/decreased serum insulin levels depending on model, improved antioxidant status, improved liver function, increased liver glycogen content, increased expression of PPARG and GLUT4 in muscles	[64,131,132,133,134,135,136,137,138,139,140,141,142,143]
*Origanum marjorana*	Marjoram	Leaves	Alpha-glucosidase, aldose reductase, DPP4, PTP1B inhibitory activity, induced activation of PPARA and PPARG;	Streptozotocin-induced diabetic rats-reduced hyperglycemia, reduced hyperlipidemia, increased/ decreased serum insulin levels depending on model, improved liver function, increased liver glycogen content, increased expression of adiponectin, lipoprotein lipase and PPARG in adipose tissue, decreased expression of leptin	[84,144,145,146,147,148,149]
*Myristica fragrans*	Nutmeg	Seed	Alpha-glucosidase, alpha-amylase, PTP1B inhibitory activity, induced PPARG and PPARA activation, increased expression of lipoprotein lipase, FAS, aP2, IRS2, CEBPA, GLUT4, CD36, CPT-1, PDK4, and acyl-CoA oxidase, stimulated phosphorylation of AMPK in myoblasts, stimulated the release of insulin from islet cells, increased phosphorylation of insulin receptor in myeloid cells	Alloxan/streptozotocin-induced diabetic rats-reduced hyperglycemia, reduced hyperlipidemia, reduced serum insulin levels, increased expression of CD36, CPT-1, PDK4, acyl-CoA oxidase, lipoprotein lipase, glycerol kinase in adipose tissue, increased expression of CPT-1, LPL, ACO and CYP4A in the liver	[49,81,150,151,152,153,154,155,156]
*Origanum vulgare*	Oregano	Leaves	Alpha-glucosidase, alpha-amylase, aldose reductase, DPP4, PTP1B inhibitory activity, induced activation of PPARG and PPARD; stimulated insulin-dependent glucose uptake in adipocytes	Alloxan/streptozotocin-induced diabetic rats-reduced hyperglycemia, reduced hyperlipidemia, increased serum insulin levels, increased liver and muscle glycogen content, reduced pancreatic alpha-amylase activity	[102,121,144,157,158,159,160,161]
*Capsicum annuum*	Paprika	Fruits	Alpha-glucosidase, alpha-amylase inhibitory activity	Alloxan-induced diabetic rats-reduced hyperglycemia, reduced hyperlipidemia	[162,163,164]
*Petroselinum crispum*	Parsley	Leaves	No significant studies identified	Streptozotocin-induced diabetic rats-reduced hyperglycemia, reduced hyperlipidemia, increased serum insulin levels, improved antioxidant status, improved liver function, increased liver and muscle glycogen content, increased liver pyruvate kinase activity	[165,166,167,168,169]
*Rosmarinus officinalis*	Rosemary	Leaves	Alpha-glucosidase, alpha-amylase, pancreatic lipase, DPP4, PTP1B inhibitory activity, induced activation of PPARG, increased glucose consumption by adipocytes, increased AMPK phosphorylation in liver cells; decreased expression of G6Pase and acetyl-CoA carboxylase B, increased expression of low-density lipoprotein receptor, SIRT1 and PPARG-coactivator 1, promoted GLUT4 translocation	Alloxan/streptozotocin-induced diabetic rats-reduced hyperglycemia, reduced hyperlipidemia, increased serum insulin levels, improved antioxidant status, improved liver function, reduced intestinal glucosidase activity, modulated activity of hexokinase, pyruvate kinase, G6Pase, FBP1, and glycogen metabolism	[40,64,68,101,144,161,170,171,172,173,174,175,176,177]
*Crocus sativus*	Saffron	Flower	Stimulated glucose uptake by skeletal muscle cells, increased phosphorylation of AMPK, increased GLUT4 translocation, induced activation of PPARA	Alloxan/streptozotocin-induced diabetic rats-reduced hyperglycemia, reduced hyperlipidemia, increased serum insulin levels, improved antioxidant status improved liver, kidney and pancreatic B-cell function	[178,179,180,181,182,183,184]
*Salvia officinalis*	Sage	Leaves	Alpha-glucosidase, alpha-amylase inhibitory activity, induced activation of PPARG, stimulated insulin-dependent glucose uptake in adipocytes	Alloxan/streptozotocin-induced diabetic rats-reduced hyperglycemia, reduced hyperlipidemia, increased serum insulin levels, improved liver and kidney function, increased GLUT4 expression	[62,102,121,185,186,187,188]
*Illicium verum*	Star anise	Fruits and seeds	Alpha-glucosidase inhibitory activity	Streptozotocin-induced diabetic rats-improved oral glucose tolerance test	[152]
*Thymus vulgaris*	Thyme	Aerial parts	Alpha-glucosidase inhibitory activity, induced activation of PPAR, stimulated glucose uptake by adipocytes and myotubes	Alloxan/streptozotocin-induced diabetic rats-reduced hyperglycemia, reduced hyperlipidemia, improved antioxidant status, improved liver and kidney functions	[102,189,190,191,192,193]
*Curcuma longa*	Turmeric	Roots	Alpha-glucosidase, alpha-amylase, aldose reductase inhibitory activity, induced activation of PPARG, stimulated insulin secretion from pancreatic cells, stimulated glucose uptake in muscle tissue	Alloxan/streptozotocin-induced diabetic rats/ KK-Ay diabetic mice-reduced hyperglycemia, reduced hyperlipidemia, increased in serum insulin levels, improved antioxidant status, improved liver function, increased activity of cholesterol-7a-hydroxylase and hepatic HMGR	[67,80,194,195,196,197,198,199,200,201,202,203,204,205,206]
*Achillea millefolium*	Yarrow	Aerial parts	Alpha-glucosidase inhibitory activity, increased expression of PPARG and GLUT4, stimulated insulin secretion by pancreatic cells	Alloxan/streptozotocin-induced diabetic rats-reduced hyperglycemia, reduced hyperlipidemia, increased serum insulin levels, improved liver and pancreas function	[207,208,209]

AceCS: acetyl-CoA synthetase 2; ACO: 1-aminocyclopropane-1-carboxylic acid oxidase; ACL: adenosine triphosphate citrate lyase; AMPK: 5’ adenosine monophosphate-activated protein kinase; CD36: cluster of differentiation 36; CEBPA: CCAAT/enhancer-binding protein alpha; CPT1: carnitine palmitoyltransferase-I; CYP4a: cytochrome P450 4A; DPP4: dipeptidyl peptidase 4; FABP4: fatty acid binding protein 4; FAS: fatty acid synthase; FBP1: fructose-1,6-bisphosphatase; FXR: farnesoid X receptor; G6Pase: glucose-6-phosphatase; GCK: glucokinase; GLP1: glucagon-like peptide 1; GLUT1/2/4: glucose transporter type 1/2/4; HMGR: 3-hyroxy-3-methyl-glutaryl-CoA reductase; IRS2: insulin receptor substrate 2; LPL: lipoprotein lipase; LXRA: liver X receptor alpha; PDX1: insulin promoter factor 1; PDK4: pyruvate dehydrogenase lipoamide kinase isozyme 4; PEPCK: phosphoenolpyruvate carboxykinase; PPARA/D/G: peroxisome proliferator-activated receptor alpha/delta/gamma; PTP1B: protein tyrosine phosphatase non-receptor type 1; SCD: stearoyl-CoA desaturase; SGLT1: sodium-glucose co-transporter-1; SIRT1: sirtuin 1; SREBP1: sterol regulatory element-binding protein 1.

**Table 2:** The major biological functions of the DIA-DB protein targets, the docking cutoff score, and the total number of potential inhibitors identified for each target.

Mode of Action	Protein Target	Function	PDB Code	Average Docking Score of Known Drugs (kcal/mol)	Docking Cutoff (kcal/mol)	Total Number of Potential Inhibitors
**Regulation of insulin secretion and sensitivity**	DPP4	Degrades and inactivates glucagon-like peptide-1 that stimulates insulin secretion from pancreas [231]	4A5S	−8.50	−9.00	260
	FFAR1	Binding of free fatty acids to receptor results in increased glucose-stimulated insulin secretion [232]	4PHU	−10.00	−10.50	6
	HSD11B1	Coverts inactive glucocorticoid precursors to active glucocorticoids; glucocorticoids counteract the effects of insulin [233]	4K1L	−9.40	−10.00	114
	INSR	Regulates glucose uptake as well as glycogen, lipid, and protein synthesis [231]	3EKN	−8.60	−9.00	47
	PTPN9	Dephosphorylates the insulin receptor, thereby reducing insulin sensitivity [234]	4GE6	−7.80	−8.00	246
	RBP4	Secreted as an adipokine that reduces insulin signaling and promotes gluconeogenesis [235]	2WR6	−7.40	−8.00	412
**Regulation of glucose metabolism**	AKR1B1	Catalyzes the reduction of glucose to sorbitol in the polyol pathway, plays a role in diabetic complications [236]	3G5E	−9.95	−10.50	96
	AMY2A	Hydrolyzes alpha-1,4-glycosidic bonds of starch during digestion of starch to glucose [237]	4GQR	−7.60	−8.00	429
	FBP1	Catalyzes the second last step in gluconeogenesis [220]	2JJK	−5.40	−6.00	210
	GCK	Phosphorylates glucose to glucose-6-phosphate for glycolysis or glycogen synthesis [234]	3IMX	−9.40	−10.00	18
	MGAM	Hydrolyzes 1,4-alpha bonds, the last step in the digestion of starch to glucose [237]	3L4Y	−6.50	−7.00	592
	PDK2	Responsible for inactivating the pyruvate dehydrogenase complex that is involved in glucose oxidation [238]	4MPC	−7.90	−8.00	190
	PYGL	Catalyzes the first step of glycogenolysis by the phosphorolysis of glycogen to glucose-1-phosphate [239]	3DDS	−8.10	−8.50	113
**Regulation of lipid metabolism**	NR5A2	Regulates the expression of genes involved in bile acid synthesis, cholesterol synthesis, and steroidogenesis [240]	4DOR	−7.50	−8.00	362
	PPARA	Regulates expression of genes involved in lipid metabolism, in particular, the oxidation of fatty acids as well as lipoprotein assembly and lipid transport [241]	3FEI	−7.60	−8.00	271
	PPARD	Regulates expression of genes involved in fatty acid catabolism [241]	3PEQ	−9.30	−10.00	60
	PPARG	Regulates expression of genes involved in adipogenesis and lipid metabolism particularly fatty acid transport, lipid droplet formation, triacyglycerol metabolism, as well as lipolysis of triglycerides [241]	2FVJ	−9.70	−10.00	75
	RXRA	Heterodimerizes with PPARs, thereby initiating gene transcription [241]	1FM9	−9.95	−10.00	24

Aldose reductase (AKR1B1), dipeptidyl peptidase-4 (DPP4), free fatty acid receptor 1 (FFAR1), fructose-1,6-bisphosphatase (FBP1), glucokinase (GCK), hydroxysteroid 11-beta dehydrogenase 1 (HSD11B1), insulin receptor (INSR), intestinal maltase-glucoamylase (MGAM), liver glycogen phosphorylase (PYGL), liver receptor homolog-1 (NR5A2), pancreatic alpha-amylase (AMY2A), peroxisome proliferator-activated receptor alpha (PPARA), peroxisome proliferator-activated receptor delta (PPARD), peroxisome proliferator-activated receptor gamma (PPARG), protein tyrosine phosphatase, non-receptor type 9 (PTPN9), pyruvate dehydrogenase kinase isoform 2 (PDK2), retinoid X receptor alpha (RXRA), and retinol binding protein 4 (RBP4).

**Table 3:** Summary of DIA-DB inverse virtual screening results for various herbs and spices.

Plant Name	Total Number of Compounds Evaluated	Total Number of Potential Anti-Diabetic Compounds (% of Total)	Compounds with 3 or More Targets
Allspice	84	41 (49%)	13
Aniseed	125	50 (40%)	23
Basil	214	58 (27%)	15
Bay leaves	179	69 (39%)	19
Black Pepper	183	84 (46%)	31
Caraway	185	43 (23%)	15
Cardamom	141	29 (21%)	2
Cinnamon	74	26 (35%)	18
Clove	147	59 (40%)	21
Cumin	146	38 (26%)	19
Dill	168	65 (39%)	27
Fennel	123	66 (54%)	42
Fenugreek	110	55 (50%)	47
Ginger	326	80 (25%)	8
Hops	98	60 (61%)	32
Lemon balm	118	53 (45%)	35
Lemongrass	132	55 (42%)	28
Liquorice	215	157 (73%)	135
Marjoram	103	31 (30%)	18
Nutmeg	96	25 (26%)	9
Oregano	177	71 (40%)	34
Paprika	166	15 (9%)	0
Parsley	78	28 (36%)	12
Rosemary	158	85 (54%)	43
Saffron	146	34 (23%)	21
Sage	162	80 (49%)	35
Star anise	69	27 (39%)	10
Thyme	204	78 (38%)	38
Turmeric	239	110 (46%)	29
Yarrow	148	72 (49%)	27

**Table 4:** Hierarchical clustering analysis of the identified bioactive compounds of various herbs and spices.

Plant	Number of Clusters	Number of Compounds in Major Clusters	Representative Compounds (Cluster Centroids)		
Allspice	6	20	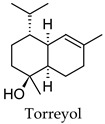		
Aniseed	13	18	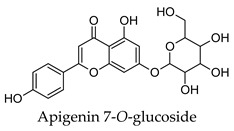		
Basil	5	50	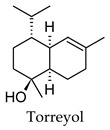		
Bay leaves	5	41; 22	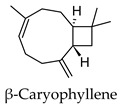		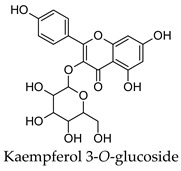
Black pepper	9	36; 24	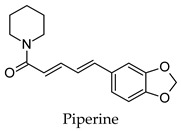		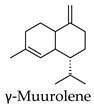
Clove	6	21; 20	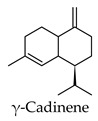		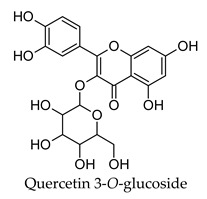
Cumin	13	10	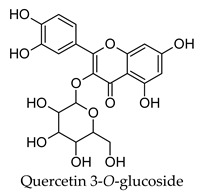		
Dill	17	10; 10	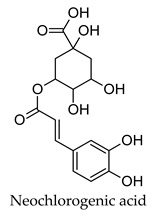		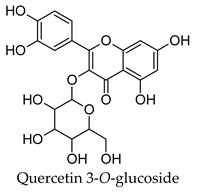
Fennel	6	52	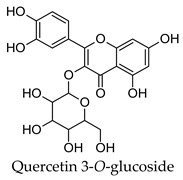		
Fenugreek	11	23; 15	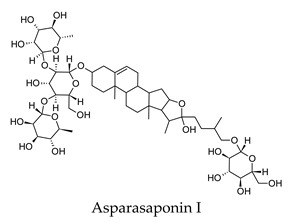		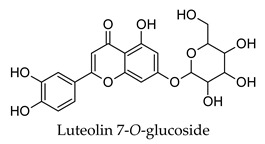
Ginger	8	51; 20	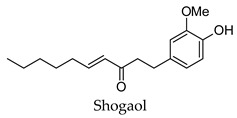	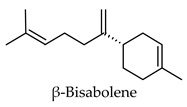	
Hops	18	19; 11	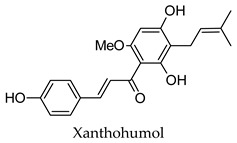	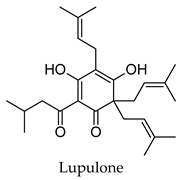	
Lemon balm	13	14; 12	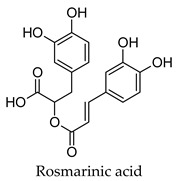	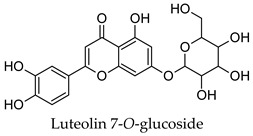	
Lemongrass	6	22; 19	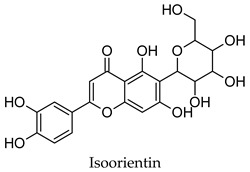	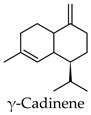	
Liquorice	12	53; 36; 30	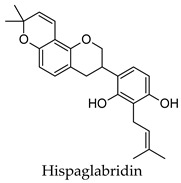	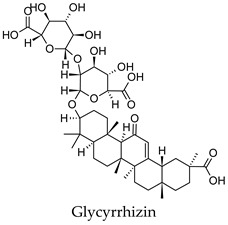	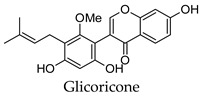
Parsley	7	11; 10	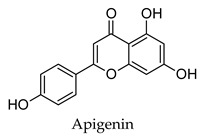	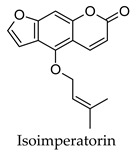	
Rosemary	20	28	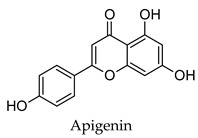		
Saffron	5	27	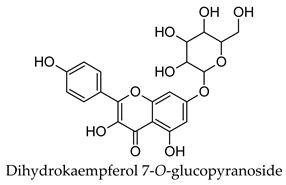		
Sage	5	39; 21	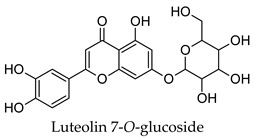	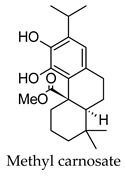	
Thyme	13	22; 21	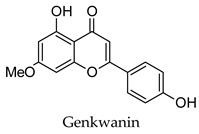	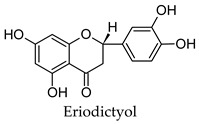	
Turmeric	25	25; 15	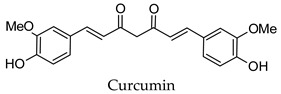	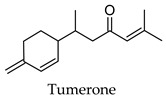	
Yarrow	10	16	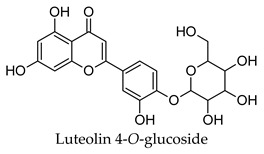		

## Supplementary Materials

The following are available online, Table S1. Docking scores of known and experimental drugs. Table S2. Protein targets and source plants of potential bioactive compounds found in herbs and spices.
