# Supplementary material for: Evaluation of the Anti-Diabetic Activity of Some Common Herbs and Spices: Providing New Insights with Inverse Virtual Screening
Source: Molecules. 2019 Nov 7;24(22):4030. doi: 10.3390/molecules24224030 (PMC6891552; doi:10.3390/molecules24224030)
Supplement: Supplementary file 1 [file molecules-24-04030-s001.pdf]

Supplementary materials

Evaluation of the anti-diabetic activity of some Common Herbs and Spices: providing new insights with Inverse Virtual Screening

Andreia S.P. Pereira [1], Antonio J. Banegas-Luna[2], Jorge Peña-García [2], Horacio Pérez-Sánchez [2,*] and Zeno Apostolides [1,*]

[1] Department of Biochemistry, Genetics and Microbiology, University of Pretoria, Pretoria Hillcrest 0083, South Africa; aspdpereira@gmail.com (A.S.P.P)

[2] Structural Bioinformatics and High-Performance Computing Research Group (BIO-HPC), Universidad Católica de Murcia, 30107, Spain; ajbanegas@alu.ucam.edu (A.J.B.-L.); Jorge.dlpg@gmail.com (J.P.-G.)

* Correspondence: hperez@ucam.edu (H.P.-S.); zeno.apostolides@up.ac.za (Z.A.); Tel: +34-968278819 (H.P.-S.); +27-12-420-2486 (Z.A.).

Table of contents	Pages
**Table S1.** Docking scores for known and experimental drugs	2-6
**Table S2.** Protein targets and source plants of potential bioactive compounds found in herbs and spices	7-67

Table S1. Docking scores for known and experimental drugs

Compound name	Docking score
**Aldose reductase**	
lidorestat	-11.4
**Crystallized ligand**	**-11.3**
minalrestat	-11.1
zenarestat	-10.9
ponalrestat	-10.6
ranirestat	-10.6
zopolrestat	-10.6
salfredin B11	-9.9
Imirestat	-9.8
Alrestatin	-9
Fidarestat	-8.9
Epalrestat	-8.7
sorbinil	-8.4
tolrestat	-8.1
**Pancreatic alpha-amylase**	
Gedunin	-9.7
Monbretin_A	-9.3
Monbretin_C	-9.2
Meliacinolin	-9.1
Azadiradione	-8.5
Fisetin	-8
AcarviostatinI03	-8
**Crystallized ligand - myricetin**	**-7.8**
Acarbose	-7.5
Caulerpenyne	-7.1
Acarviosine_glucose	-7
AcarviostatinI01	-6.7
Ethyl-caffeate	-6.3
Miglitol	-5.3
Metformin	-4.5
**Dipeptidyl peptidase 4**	
**Crystallized ligand**	**-10.5**
Sitagliptin	-9.2
Linagliptin	-8.9
Gosogliptin	-8.9
Berberine	-8.5
Omarigliptin	-8.4
Saxaglitpin	-8.3
Anagliptin	-8.2
Vildagliptin	-7.9
Alogliptin	-7.7
Trelagliptin	-7.2
**Fructose-1,6-bisphosphatase 1**	
1fta	-6.4
4mjo	-6.3

2y5k	-6.1
2fix	-5.8
2y5l	-5.8
**Crystallized ligand**	-5.3
3kc1	-5.3
MDL-29951	-5.2
Managlinat dialanetil	-4.9
MB07803	-4.9
3kc0	-4.8
3kbz	-4.7
2vt5	-4.3
**<u>Free fatty acid receptor 1</u>**	
AM1638	-11.3
MR1704	-11.1
LY-2881835	-11
AMG837	-10.7
TUG-770	-10.4
5tzr	-10.3
GW9508	-10.1
Fasiglifam	-10
P-1736	-10
**Crystallized ligand - TAK-875**	**-9.8**
PBI-4050	-8.7
GW1100	-7.6
**<u>Glucokinase</u>**	
**Crystallized ligand**	**-10.6**
Piragliatin	-10.4
4no7	-10.2
PF04937319	-9.7
LY2599506	-9.6
PF0499153	-9.3
AM2394	-9.3
5v4w	-9.2
Ro-28-1675	-9.1
AMG151	-8.9
AMG3969	-8.8
LY2608204	-8.3
AZD1656	-8.3
**<u>11B-Hydroxysteroid dehydrogenase type 1</u>**	
Enoxolone	-11
ABT-384	-10.8
Carbenoxolone	-10.8
EGCG	-9.8
RO5093151	-9.8
Acetoxolone	-9.7
11-beta-hydroxyprogesterone	-9.5
INCB13739	-9.3
11-beta-hydroxyandrostenedione	-9.1

Curcumin	-8.7
11-beta-hydroxytestosterone	-8.6
18-alpha-glycyrrhizic acid	-8.4
Cortisol	-8.3
**Crystallized ligand**	**-8.3**
**Insulin receptor**	
linsitinib	-9.7
GSK1904529A	-9.4
3ekk	-9.4
5e1s	-8.7
**Crystallized ligand**	**-8.7**
Ceritinib	-8.6
NVP-AEW541	-8.5
BMS-536924	-8.4
4IBM	-8.4
GSK1838705A	-8.2
BMS-754807	-7.7
AGL-2263	-7.4
**Intestinal maltase-glucoamylase**	
Cyanidin	-8.7
Epigallocatechin gallate	-8.4
Meliacinolin	-8.0
terretonin	-7.9
territrem_A	-7.9
terretonin_A	-7.7
acarbose	-6.8
nojirimycin	-6
de-O-sulfonated-kotalanol	-5.9
kotalanol	-5.9
**Crytallized ligand – NR4-8II**	**-5.9**
neoponkoranol	-5.8
1-deoxynojirimycin (duvoglustat)	-5.4
ponkoranol	-5.4
voglibose	-5.1
salacinol	-5.0
BJ2661	-4.9
casuarine	-4.7
miglitol	-4.5
valiolamine	-4.4
**Liver receptor homolog-1**	
ML179	-8.8
ML180	-8.2
Cpd3	-8
RJW100 alcohol derivative 2N	-7.8
RJW100	-7.7
GSK8470	-7.6
RJW100 alcohol derivative 6N	-7.4
RJW100 alcohol derivative 5N	-7

**Crystallized ligand**	**-6.5**
DLPC	-5.6

<u>Pyruvate dehydrogenase kinase 1</u>

5m4p	-9.4
4v25	-9.3
5j6a	-8.7
**Crystallized ligand**	**-7.8**
5j71	-7.7
4mpe	-7.5
4mpn	-7.5
AZD-7545	-7.4
CPI-613	-6.8
5m4k	-6.7

<u>Peroxisome proliferator-activated receptor alpha</u>

CP775146	-8.6
Lobeglitazone	-8.5
**Crystallized ligand**	**-8.3**
GW6471	-8.1
GW7647	-7.9
Bezafibrate	-7.7
Fenofibrate	-7.7
Pirinixic acid	-7.5
MK886	-7.0
Elafibranor	-6.4
Oleyethanolamide	-6.2

<u>Peroxisome proliferator-activated receptor delta</u>

Telmisartan	-11.8
**Crystallized ligand**	**-11.3**
Sodelglitazar	-10
GW0742	-9.7
GW501506	-9.4
GSK3787	-9.2
GSK-0660	-8.7
MBX-8025	-8.6
Indeglitazar	-8.5
GFT-505	-8.4
L-165041	-7.0

<u>Peroxisome proliferator-activated receptor gamma</u>

Farglitazar	-10.9
Muraglitazar	-10.8
Balaglitazone	-10.5
Imiglitazar	-10.5
**Crystallized ligand**	**-10**
FK-614	-9.5
Pioglitazone	-9.4
RWJ-348260	-8.9
Rosiglitazone	-8.8

Ciglitazone	-8.8
Metaglidasen	-8.5
<u>**Protein tyrosine phosphatase N9**</u>	
Chebulinic acid	-9
**Crystallized ligand**	**-7.7**
4ge5	-7.4
4ge2	-7.1
<u>**Liver glycogen phosphorylase**</u>	
3dd1	-9.7
**Crystallized ligand**	**-9.6**
3ddw	-9.2
3-O-acyl oleanolic acid derivative 4	-8.8
Oleanolic acid	-8.1
CP-91149	-8
3ceh	-7.7
2ati	-7.6
N-Acetyl-β-D-glucopyranosylamine derivative 10	-7.6
N-Acyl-N'-β-D-glucopyranosyl urea derivative 12	-7.4
N-β-D-Glucopyranosyl monoamide of dicarboxylic acid derivative 2	-6.7
β-D-Glucopyranosyl Nucleosides derivative 2	-6.6
<u>**Retinol binding protein 4**</u>	
Tretinoin	-9
4psq	-8.6
**Crystallized ligand**	**-7.9**
2-((3aR,5r,6aS)-5-(2-(trifluoromethyl)phenyl)octahydrocyclopenta[c]pyrrole-2-carboxamido)benzoic acid	-6.7
A1120	-6.6
4o9s	-6.5
BPN-14136	-6.4
<u>**Retinoic X receptor alpha**</u>	
Bexarotene	-13
**Crystallized ligand**	**-10.6**
5tbp	-10.2
CD3254	-10.1
PA 451	-9.4
UVI 3003	-9.4
4zsh	-8.9
Magnolol	-8.7
HX 531	-8.5
AGN34	-8.5
Danthron	-7.8

Table S2. Protein targets and source plants of potential bioactive compounds found in herbs and spices

**Compound**	**Enzyme**	**Source plants**
(-)-illicinone A	RBP4	Star anise
(+)-aromadendrene	NR5A2; RBP4	Allspice; bay leaves; cardamom; ginger; oregano; paprika; sage; thyme
(1R,2R)-1'-(4-hydroxyphenyl)propane-1,2-diol-2-O-B-D-glucopyranoside	MGAM	Aniseed
(1R,2R)-Guaiacyl-glycerol-3'-O-B-D-glucopyranoside	MGAM	Aniseed
(1R,5R,6S,7S,9S,10S,11R)-1,9-dihydroxyeudesm-3-ene-12,6-olide-9-O-B-D-glucopyranoside	AMY2A	Cumin
(1S,2S)-1'-(4-hydroxyphenyl)propane-1,2-diol-2-O-B-D-glucopyranoside	MGAM	Aniseed
(1S,2S)-anthole-glycol-2-O-B-D-glucopyranoside	MGAM	Aniseed
(2E)-piperamide-C5:1	NR5A2; RBP4	Black pepper
(2E,4E,8E)-piperamide-C9:3	AKR1B1; MGAM; NR5A2; PPARA; RBP4; RXRA	Black pepper
(2E,6E)-piperamide-C7:2	AKR1B1; NR5A2; PPARA; RBP4	Black pepper
(2E,8E)-piperamide-C9:2	AKR1B1; NR5A2; RBP4; RXRA	Black pepper
(2-hydroxy-3-methoxy-5-allyl)phenyl-B-D-(6-O-E-sinapoyl)glucopyranoside	MGAM	Allspice
(2R,3R)-3,4',7-trihydroxy-3'prenyl-flavanone	AMY2A; DPP4; MGAM; NR5A2; PPARA; PTPN9; RBP4	Liquorice
(3-(cyclopent-2-enyl)-2-methylprop-1-ene-1,1-diyl)dibenzene	MAGAM; NAR5A2; RBP4	Basil
(3-(phenylsulfinylmethyl)cyclopropane-1,2-diyl)dibenezene	AKR1B1; DPP4; MGAM; NR5A2; PPARA; PPARD; RBP4	Basil
(3b,5a,8a,22E) 5,8-epidioxy-ergosta-6,22-dien-3-ol	AMY2A; GCK; HSD11B1; MGAM; NR5A2; PPARD	Fennel

(3R)-2',3',7-trihydroxy-4'-methoxyisoflavan	AMY2A; MGAM; NR5A2; RBP4	Liquorice
(3R)-vestitol	MGAM; RBP4	Liquorice
(4S)-p-menth-1-ene-7,8-diol 8-O-b -D-glucopyranoside	RBP4	Caraway
(4S,5S)-germacrone-4,5-epoxide	RBP4	Turmeric
(6E)-piperamide-C7:1	AKR1B1; MGAM; RBP4	Black pepper
(6R)-[(1R)-1,5-dimethylhex-4-enyl]-3-methylcyclohex-2-en-1-one	RBP4	Turmeric
(6S)-2-methyl-6-[(1R,5S)-(4-methene-5-hydroxyl-2-cyclohexen)-2-hepten-4-one	RBP4	Turmeric
(7H)-6,9a-methano-4H-cyclopenta[9,10]cyclopropa[5,6]cyclodeca[1]	NR5A2	Ginger
(7S)-8'-(4'-hydroxy-3'-methoxyphenyl)-7-hydroxypropyl)benzene-2,4-diol	RBP4	Nutmeg
(7S)-8'-(benzo[3',4']dioxol-1'-yl)-7-hydroxypropyl)benzene-2,4-diol	RMGAM; NR5A2; PPAPA; RBP4	Nutmeg
(8R,8S)-7-(4-hydroxy-3-methoxyphenyl)-8'-methylbutan-8-yl)-3'-methoxybenzene-4',5'-diol	RBP4	Nutmeg
(E)-caryophyllene	RBP4	Allspice; basil; bay leaves; caraway; clove; dill; ginger; lemongrass; marjoram; paprika; rosemary; sage; thyme; turmeric; yarrow
(E)-3'-hydroxy-4'-(1''-hydroxyethyl)-phenyl-4-methoxycinnamate	RBP4	Basil
(E)-a-atlantone	RBP4	Dill; turmeric
(E)-b-Ionone	RBP4	Basil; lemongrass; paprika; saffron
(E)-g-atlantone	RBP4	Turmeric
(E)-sesquisabinene hydrate	RBP4	Turmeric
(E,E)-a-farnesene	RBP4	Clove; ginger; lemongrass; oregano; sage; turmeric
(E,E,E)-3,7,11,15-tetramethylhexadeca-1,3,6,10,14-pentaene	RBP4	Turmeric
(R)-butyl-rosmarinate	MGAM; PPARA	Oregano
(Z)-a-atlantone	RBP4	Turmeric
(Z)-a-trans-bergamotol acetate	RBP4	Lemon balm
(Z)-g-atlantone	RBP4	Turmeric
1-(2',4'-dihydroxyphenyl)-2-hydroxy-3-(4''-hydroxyphenyl)-1-propanone	RBP4; MGAM; PPARA	Liquorice
1-(3-cyclopentylpropyl)-2,4-dimethy-benzene	RBP4	Turmeric

1-(4'-hydroxy-3',5'-dimethoxyphenyl)-7-(4'-hydroxy-3'-methoxyphenyl)-4-hepten-3-one	MGAM; PPARA	Ginger
1-(4-hydroxy-3-methoxyphenyl)-2,4-dehydro-6-decanone	MGAM	Ginger
1-(4-hydroxy-3-methoxyphenyl)-5-(4-hydroxyphenyl)-1, 4-pentadiene-3-one	MGAM	Turmeric
1-(4-hydroxy-3-methoxyphenyl)-7-(3, 4-dihydroxyphenyl)-1, 6-heptadiene-3, 5-dione	AKR1B1; AMY2A; MGAM; PPARA; RBP4	Turmeric
1-(4'-hydroxy-3'-methoxyphenyl)-7-dodecen-3-one	RBP4	Ginger
1-(4-hydroxyphenyl)-7-(3, 4-dihydroxyphenyl)-1, 6-heptadiene-3, 5-dione	AKR1B1; AMY2A; MGAM; PPARA; RBP4	Turmeric
1-(4'-methoxyphenyl)-(1R,2R)-propan-1-ol-2-O-D-glucopyranoside	RBP4	Star anise
1-(4'methoxyphenyl)-(1S,2R)-propan-1-ol-2-O-D-glucopyranoside	RBP4	Star anise
1-(4'-methoxyphenyl)-(1S,2S)-propan-1-ol-2-O-D-glucopyranoside	RBP4	Star anise
1-(9-hydroxy-4-methylethyl)-benzoic-acid	MGAM	Cumin
1,10-dehydro-10-deoxy-9-oxozedoarondiol	RBP4	Turmeric
1,2-diphenylcyclobutane	MGAM; NR5A2; RBP4	Basil
1,3-dicaffeoylquinic acid	AMY2A; MGAM; NR5A2; PTPN9	Fennel; yarrow
1,4-dicaffeoylquinic acid	AMY2A; DPP4; FBP1; MGAM; NR5A2; PTPN9; PYGL	Dill; fennel; yarrow
1,5-bis(4-hydroxy-3-methoxyphenyl)-1,4-pentadien-3-one	AMY2A; MGAM	Turmeric
1,5-bis(4-hydroxyphenyl)-penta-(1E,4E)-1,4-dien-3-one	MGAM; RBP4	Turmeric
1,5-dicaffeoylquinic acid	AMY2A; DPP4; MGAM; NR5A2; PPARA	Fennel; yarrow
1,5-dihydroxy-1-(4-hydroxy-3-methoxyphenyl)-7-(4-hydroxyphenyl)-4,6-heptadiene-3-one	MGAM; PPARA; RBP4	Turmeric
1,5-dihydroxy-1-(4-hydroxyphenyl)-7-(4-hydroxy-3-methoxyphenyl)-4,6-heptadiene-3-one	MGAM; PPARA; RBP4	Turmeric
1,5-dihydroxy-1,7-bis(4-hydroxy-3-methoxyphenyl)-4,6-heptadiene-3-one	MGAM; PPARA; RBP4	Turmeric
1,5-dihydroxy-1,7-bis(4-hydroxyphenyl)-4,6-heptadiene-3-one	MGAM; PPARA; RBP4	Turmeric
1,5-epoxy-3-carbonyl-1,7-bis(4-hydroxyphenyl)-4,6-heptadiene	AKR1B1; AMY2A; MGAM; NR5A2; PTPN9; RBP4	Turmeric

1,7-bis(4-hydroxy-3-methoxyphenyl)-1,4,6-heptatrien-3-one	AKR1B1; MGAM	Turmeric
1,7-bis-(4'-hydroxy-3'-methoxyphenyl)-3,5-heptadione	RBP4	Ginger
1,7-bis-(4'-hydroxy-3'-methoxyphenyl)-3-hydroxy-acetoxyheptane	MGAM	Ginger
1,7-bis-(4'-hydroxy-3'-methoxyphenyl)-4-heptene-3-one	MGAM; PPARA; RBP4	Ginger
1,7-bis-(4'-hydroxy-3'-methoxyphenyl)-5-methoxyheptan-3-one	RBP4	Ginger
1,7-bis(4-hydroxyphenyl)-1,4,6-heptatrien-3-one	AKR1B1; AMY2A; MGAM	Turmeric
1,7-bis(4-hydroxyphenyl)-1-heptene-3,5-dione	AKR1B1; MGAM; RBP4	Turmeric
1-[(2E,4E)-2,4-dodecadienoyl]pyrrolidine	RBP4	Black pepper
10-epigazaniolide	NR5A2	Bay leaves
10-gingerdiol	RBP4	Ginger
10-gingerdione	RBP4	Ginger
10-shogaol	RBP4	Ginger
11,12,20-trihydroxy-abieta-8,11,13-triene	MGAM	Sage
11,13-dehydrosantonin	NR5A2; RBP4	Bay leaves
11-paradol	RBP4	Ginger
12-hydroxy-20-norabieta-8,11,13-triene	MGAM	Sage
12-methoxy-1-oxo-20-norabieta-8,11,13-triene	RBP4	Sage
12-shogaol	RBP4	Ginger
13-cis-crocetin	AMY2A; DPP4	Saffron
18a-glycyrrhizin	AMY2A; DPP4; FBP1; INSR; MGAM; PDK2; PTPN9; PYGL	Liquorice
1-Caffeoylquinic acid	MGAM; PTPN9; RBP4	Fennel
1-cinnamoylpiperidine	MGAM; RBP4	Black pepper
1-dehydro-3-gingerdione	RBP4	Ginger
1-dehydro-6-gingerdione	RBP4	Ginger
1-dehydro-6-gingerol	RBP4	Ginger
1-dehydro-8-gingerdione	RBP4	Ginger

1-hydroxy-1,7-bis(4-hydroxy-3-methoxyphenyl)-6-heptene-3,5-dione	AKR1B1; AMY2A; MGAM; PPARA; PTPN9	Turmeric
1-hydroxymethyl-B-methyl-benzenethanol	MGAM	Cumin
1-hydroxypinoresinol1-B-D-glucopyranoside	AMY2A; MGAM	Sage
1-O-(4-hydroxybenzoyl)-B-D-glucopyranose	RBP4	Rosemary
1-oxomiltirone	RBP4	Sage
2''-rhamnosylisovitexin	AMY2A; DPP4; FBP1; INSR; MGAM; NR5A2; PDK2; PTPN9; PYGL	Bay leaves
2-(2'-methyl-1'-propenyl)-4, 6-dimethyl-7-hydroxyquinoline	MGAM; RBP4	Turmeric
2-(4-hydroxy-3-methoxyphenyl)-ethyl-O-B-D-glucopyranoside	MGAM; PTPN9; RBP4	Bay leaves
2,2,4-trimethyl-3-(3,8,12,16-tetramethyl-heptadeca-3,7,11,15-tetraenyl)-cyclohexanol	MGAM; PPARA; PPARG	Turmeric
2,2'-oxybis[octahydro-7,8,8-trimethyl-4,7-methanobenzofuran	AMY2A; HSD11B1; MGAM; NR5A2; PDK2	Turmeric
2,3',4,4',a-pentahydroxy-3,5'-diprenyl-dihydrochalcone	AMY2A; FBP1; HSD11B1; MGAM; NR5A2; PPARA; PPARG	Liquorice
2,3',4,4',a-pentahydroxy-3-prenyl-dihydrochalcone	AKR1B1; AMY2A; FBP1; MGAM; NR5A2; PPARA; RXRA; RBP4	Liquorice
2,3',4,4'-tetrahydroxy-3,5'-diprenylchalcone	AMY2A; DPP4; MGAM; NR5A2; PDK2; PPARA; PPARG	Liquorice
2',3,4'-trihydroxy-3'-g,g-dimethylallyl-6'',6''-dimethylpyrano[2'',3'':4,5]chalcone	AMY2A; DPP4; HSD11B1; MGAM; NR5A2; PPARA; PPARD; PPARG; PTPN9	Liquorice

2',3-dihydroxy-4'-methoxy-3",3"-dimethylpyrano[2",3":7,8]isoflavanone	AMY2A; FBP1; HSD11B1; MGAM; NR5A2; PPARA	Liquorice
2,4-decadienoic acid piperidine	RBP4	Black pepper
2,6,11,15-tetramethyl-hexadeca-2,6,8,10,14-pentaene	RBP4	Turmeric
2,8-epoxy-5-hydroxybisabola-3,10-diene-9-one	RBP4	Turmeric
20-hydroxyferruginol	MGAM; RBP4	Sage
20-oxopregn-16-en-12-yl acetate	AMY2A; HSD11B1; NR5A2	Turmeric
22-acetoxylglycyrrhizin	AMY2A; DPP4; FBP1; MGAM; PDK2; PTPN9; PYGL	Liquorice
23-Monosulfate ester of 2a,23-dihydroxyurs-12-ene-28-oic acid 3-O-β-Dglucopyranoside	AMY2A; NR5A2	Lemon balm
24-Methylene cycloartenol	AKR1B1; AMY2A; DPP4; HSD11B1; MGAM; NR5A2; PDK2; PPARG	Caraway
2-C-methyl-D-erythritol-1-O-B-D-(6-O-4-hydroxybenzoyl)glucopyranoside	MGAM; PTPN9	Aniseed
2-C-methyl-D-erythritol-1-O-B-D-(6-O-4-methoxybenzoyl)glucopyranoside	MGAM; PTPN9	Aniseed
2-hydroxymethyl-benzoate	FBP1	Basil
2-methoxy-5-hydroxybisabola-3,10-diene-9-one	RBP4	Turmeric
2-methyl-6-(4-hydroxy-3-methylphenyl)-2-hepten-4-one	RBP4	Turmeric
2-methyl-6-(4-hydroxyphenyl)-2-hepten-4-one	RBP4	Turmeric
2-methylcortisol	MGAM	Ginger
2-methylene-5a-cholestan-3b-ol	AMY2A; MGAM	Ginger
2-Phenylethyl phenyl acetate	RBP4	Clove; rosemary
3-(3,4-dihydroxyphenyl)lactic acid	MGAM	Lemon balm
3-(4-hydroxy-3-methoxyphenyl)propane-1,2-diol-2-O-B-D-(6-O-galloyl)glucopyranoside	MGAM; PDK2; PTPN9	Allspice
3'-(R)-MTPa ester	AKR1B1; AMY2A; MGAM; NR5A2; PPARA; PTPN9; RBP4	Fennel

3'-(S)-MTPa ester	AKR1B1; AMY2A; NR5A2; PPARA; PTPN9; RBP4	Fennel
3,23-Disulfate ester of 3b,19a,23-trihydroxyurs-12-en-28-oic acid	MGAM	Lemon balm
3,3',4,4'-tetrahydroxy-2'-methoxy-5-prenylchalcone	AKR1B1; AMY2A; MGAM; PPARA; RBP4	Liquorice
3,3',4'-tri-O-methylellagic-acid	RBP4	Clove
3,4-dicaffeoylquinic acid	AKR1B1; AMY2A; DPP4; HSD11B1; MGAM; NR5A2; PDK2; PPARA; PTPN9; PYGL	Lemongrass; yarrow
3,4-dihydroxyphenylacetic acid	MGAM	Thyme
3,4-dimethoxy-3,4-desmethylenedioxycubebin	AKR1B1; AMY2A; PPARA	Black pepper
3,4-seco-(24Z)-cycloart-4(28),24-diene-3,26-dionic acid,26-methyl ester	MGAM; NR5A2	Star anise
3,5-diacetoxy-7-(4-hydroxy-3-methoxyphenyl)-1-(3,4-dihydroxy-5-methoxyphenyl)heptane	MGAM	Ginger
3,5-dicaffeoylquinic_acid	ARB1B1; AMY2A; DPP4; MGAM; NR5A2; PDK2	Aniseed; dill; lemongrass; yarrow
3,5-dihydroxy-1-(4-hydroxy-3,5-dimethoxyphenyl)-7-(4-hydroxy-3-methoxyphenyl)heptane	MGAM	Ginger
3,5-dihydroxy-1,7-bis(4-hydroxy-3-methoxyphenyl)heptane	MGAM; RBP4	Ginger
3a-acetoxyeudesma-1,4(15),11(13)-trien-12,6a-olide	NR5A2	Bay leaves
3-acetoxy-3-dihydrodemethoxy-6-shogaol	RBP4	Ginger
3-acetoxy-5-hydroxy-1-(4-hydroxy-3-methoxyphenyl)-7-(3,4-dihydroxyphenyl)heptane	MGAM; PPARA	Ginger
3-dihydro-6-demethoxyshogaol	RBP4	Ginger
3-gentiobiosylkaempferol	AMY2A; FBP1; MGAM; NR5A2; PPARA; PTPN9	Saffron
3'-geranylchalconaringenin	AKR1B1; MGAM; NR5A2; PPARA	Hops

3-hydroxy-1,7-bis-(4-hydroxyphenyl)-6-heptene-1,5-dione	AKR1B1; PPARA; PTPN9; RBP4	Turmeric
3'-hydroxy-4'-O-methylglabridin	AMY2A; DPP4; FBP1; MGAM; NR5A2	Liquorice
3-hydroxyglabrol	AMY2A; MGAM; NR5A2; PDK2; PPARA; PPARG; PTPN9; PYGL; RBP4; RXRA	Liquorice
3-methoxyapigenin	AMY2A; MGAM; NR5A2; RBP4	Oregano
3-methoxyglabridin	AMY2A; DPP4; MGAM; NR5A2	Liquorice
3-O-p-coumaroylquinic acid	MGAM; PPARA; PTPN9	Cumin; dill
3-oxoeudesma-1,4,11(13)-trien-12,6a-olide	NR5A2; RBP4	Bay leaves
4''-(4'''-hydroxyphenyl-3-methoxy)-2''-oxo-3''-butenyl-3-(4'-hydroxyphenyl)-propenoate	AKR1B1; MGAM; RBP4	Turmeric
4-(1H-1,2,3,4-tetrazol-1-yl)-benzene-acetic-acid	RBP4	Ginger
4-(3,4-dihydroxybenzoyloxymethyl)-phenyl-B-D-glucopyranoside	AMY2A; MGAM; PPARA	Oregano
4, 5-dihydroxybisabola-2,10-diene	RBP4	Turmeric
4,5-dicaffeoylquinic acid	AKR1B1; AMY2A; DPP4; FBP1; MGAM; NR5A2; PDK2; PPARA; PTPN9; PYGL	Aniseed; lemongrass; yarrow
4,5-dihydropiperine	AKR1B1; NR5A2; PPARA; RBP4	Black pepper
4',7-dihydroxyflavone	AMY2A; MGAM; NR5A2; PPARA; RBP4	Liquorice
4,8,12,16-tetramethylheptadecam-4-olide	RBP4	Saffron
4-[[(2',5'-Dihydroxybenzoyl)oxy]methyl]phenyl O-b-D-glucopyranoside	AMY2A; MGAM; PPARA	Oregano

4-[[(3',4'-Dihydroxybenzoyl)oxy]methyl] phenyl O-b-D-[6-O-(3",5"-dimethoxyl-4"-hydroxybenzoyl)] glucopyranoside	AMY2A; DPP4; FBP1; MGAM; NR5A2; PPARA; PTPN9	Oregano
4-allyl-2-(3-methylbut-2-enyl)-1,6-methylenedioxybenzene-3-ol	RBP4	Star anise
4-allylphenol	MGAM	Allspice; basil; clove
4'-carbomethoxy-2'-hydroxyphenyl-ferulate	MGAM	Basil
4'-geranyloxyferulic acid (GOFa)	MGAM; RBP4	Aniseed; dill
4-hydroxyacetophenone-4-O-B-D-apiofuranosyl-(1-6)-O-B-D-glucopyranoside	PTPN9	Sage
4-hydroxybisabola-2,10-diene-9-one	RBP4	Turmeric
4-hydroxyglabrin	AMY2A; FBP1; MGAM; NR5A2; PDK2; PPARA	Liquorice
4-hydroxymethylbenzoic acid	FBP1	Basil
4-Methoxy-5-hydroxybisabola-2,10-diene-9-one	RBP4	Turmeric
4-methylene-5-hydroxybisabola-2,10-diene-9-one	RBP4	Turmeric
4-Methylstilbene	MGAM; NR5A2; RBP4	Dill
4-O-B-D-glucopyranosyloxybenzoic acid	PPARA; RBP4	Aniseed
4-O-feruloylquinic acid	MGAM	Lemongrass
4'-O-methyl-3'-prenylchalconaringenin	AKR1B1; MGAM; PPARA; RXRA	Hops
4'-O-methylglabridin	AKR1B1; AMY2A; DPP4; FBP1; HSD11B1; MGAM; NR5A2; PDK2	Liquorice
4-O-methylguaiacyl-glycerol-2'-O-B-D-glucopyranoside	MGAM	Aniseed
4-O-p-coumaroylquinic acid	FBP1; MGAM; PTPN9	Cumin; dill; fennel
4-vinylsyringol	AMY2A; DPP4; FFAR1; MGAM; NR5A2; RBP4	Ginger
5-(5-carboxymethyl-2-oxocyclopentyl)-3Z-pentenyl-B-D-(6-O-galloyl)glucopyranoside	DPP4; MGAM; NR5A2; PDK2; PPAPAR; PTPN9; PYGL	Allspice
5,10(15)-Cadienen-4-ol	RBP4	Black pepper

5,4'-dihydroxy-6,7,8-trimethoxyflavone	RBP4	Thyme
5,4'-dihydroxy-6,7-dimethoxyflavone	AMY2A; RBP1; MGAM; NR5A2; PDK2; PPARA; PTPN9; RBP4	Thyme
5,4'-dihydroxy-7-methoxyflavone	AMY2A; MGAM; RBP4	Thyme
5,6-diepikarpoxanthin (Carpoxanthin)	MGAM; NR5A2	Paprika
5-[4" -hydroxy-3 "-methyl-2" -butenyloxy]-6,7-furocoumarin	MGAM; RBP4	Dill
5-Coumaroylquinic acid	MGAM; PPARA; RBP4	Fennel
5-epi-7-epi-a-eudesmol	RBP4	Lemongrass
5'-formylglabridin	AKR1B1; AMY2A; DPP4; FBP1; HSD11B1; MGAM; NR5A2; PPARA	Liquorice
5-hydroxy-1-(3,4-dihydroxy-5-methoxyphenyl)-7-(4-hydroxy-3-methoxyphenyl)-3-heptanone	MGAM; PPARA	Ginger
5-hydroxy-1-(4-hydroxy-3-methoxyphenyl)-7-(3,4-dihydroxyphenyl)-3-heptanone	MGAM; PPARA; RBP4	Ginger
5-hydroxy-1,7-bis(4-hydroxy-3-methoxyphenyl)-3-heptanone	RBP4	Ginger
5-hydroxy-7,4'-dimethoxyflavone	AMY2A; FBP1; MGAM; NR5A2; PDK2; PTPN9	Thyme
5-hydroxyl-1-(4-hydroxy-3-methoxyphenyl)-7-(4-hydroxyphenyl)-4,6-heptadiene-3-one	AMY2A; MGAM; RBP4	Turmeric
5-hydroxyl-1,7-bis(4-hydroxy-3-methoxyphenyl)-4,6-heptadiene-3-one	PPARA	Turmeric
5-hydroxyl-7-(4-hydroxy-3-methoxyphenyl)-1-(4-hydroxyphenyl)-4,6-heptadiene-3-one	AKR1B1; RBP4	Turmeric
5-hydroxyl-ar-turmerone	RBP4	Turmeric
5'-methoxylicarin-B	PPARA	Nutmeg
5-methoxypsoralen (bergapten)	RBP4	Fennel; parsley
5-O-methylglycyrol	AMY2A; MGAM; NR5A2; PPARD	Liquorice
6"-acetylapiin	AMY2A; DPP4; FBP1; MGAM; NR5A2; PDK2; PTPN9	Parsley

6''-O-(E)-coumaroylnepitrin	PAMY2A; DPP4; FBP1; INSR; MGAM; NR5A2; PDK2; PPARA; PPARG; PTPN9; PYGL	Rosemary
6''-O-(E)-feruloylglucopyranoside	MGAM	Sage
6''-O-(E)-feruloylhomoplantaginin	AMY2A; DPP4; FBP1; MGAM; NR5A2; PDK2; PPARA	Rosemary
6''-O-(E)-feruloylnepitrin	AMY2A; DPP4; FBP1; MGAM; NR5A2; PDK2; PPARA; PTPN9	Rosemary
6, 8-C-dihexosylapigenin	AMY2A; DPP4; HSD11B1; MGAM	Fennel
6,7-dimethoxyrosmanol	MGAM	Sage
6,8-diprenylnaringenin	AKR1B1; AMY2A; DPP4; MGAM; NR5A2; PDK2; PPARA	Hops
6-acetyl-5-hydroxy4-methylcoumarin (Liqcoumarin)	RBP4	Liquorice
6-C-B-D-(6'-O-galloyl)glucosylnoreugenin	AMY2A; DPP4; FBP1; HSD11B1; MGAM; NR5A2; PTPN9; PYGL	Clove
6-C-pentosyl-8-C-hexosyl apigenin	DPP4	Lemongrass
6-C-pentosyl-8-C-hexosyl luteolin	AMY2A; DPP4; FBP1; NR5A2	Lemongrass
6-C-pentosyl-luteolin	AMY2A; FBP1; MGAM; PDK2; PPARA	Lemongrass
6-Epidesacetyllaurenobiolide	NR5A2	Bay leaves
6-geranylnaringenin	AKR1B1; AMY2A; FBP1; HSD11B1; MGAM; NR5A2; PDK2; PPARA	Hops

6-gingerdiol acetaldehyde acetal	RBP4	Ginger
6-gingerdiol geranial acetal	MGAM; PPARA	Ginger
6-hydroxy-10-shogaol	RBP4	Ginger
6-hydroxy-6-shogaol	RBP4	Ginger
6-hydroxy-6-shogaol	RBP4	Ginger
6-hydroxy-8-shogaol	RBP4	Ginger
6-hydroxyluteolin-7-O-glucoside	AMY2A; DPP4; FBP1; HSD11B1; MGAM; PDK2; PPARA; PTPN9	Rosemary; sage; yarrow
6-hydroxyluteolin-7-O-glucuronide	AMY2A; DPP4; FBP1; HSD11B1; MGAM; PDK2; PPARA; PTPN9	Sage
6-paradoldiene	RBP4	Ginger
6-prenylnaringenin	AMY2A; MGAM; NR5A2; PPARA	Hops
6-shogaol	RBP4	Ginger
7,4-dimethylapigenin	RBP4	Thyme
7,8-dihydroxy-4'-methoxy-6-prenylisoflavanone	AMY2A; DPP4; MGAM; PPARA; PTPN9	Liquorice
7-a-hydroxy manool	RBP4	Dill
7-a-hydroxycampesterol	DPP4; HSD11B1; MGAM; NR5A2; PPARD	Fennel
7-epi-a-selinene	RBP4	Bay leaves; sage
7-epi-sesquithujene	RBP4	Turmeric
7-gingerol	RBP4	Ginger
7-hydroxycarveol-7-O-B-D-glucopyranoside	MGAM; RBP4	Caraway
7-isopentenyloxycoumarin	RBP4	Aniseed; dill
7-methoxyrosmanol	MGAM	Sage
7-methylepirosmanol	MGAM	Rosemary

8,8-dimethyl-3,4-dihydro-2H,8H-pyrano[2,3-f]-chromon-3-ol	RBP4	Liquorice
8-C-B-D-(6'-O-galloyl)glucosylnoreugenin	AMY2A; DPP4; FBP1; MGAM; NR5A2; PPARA; PTPN9; PYGL	Clove
8'-geranylnaringenin	AKR1B1; AMY2A; DPP4; GCK; MGAM; NR5A2; PDK2; PPARA	Hops
8-hydroxymethyl-8-methyl-3,4-dihydro-2H,8H-pyrano[2,3-f]-chromon-3-ol	RBP4	Liquorice
8-paradol	RBP4	Ginger
8-paradoldiene	RBP4	Ginger
8-prenylnaringenin	AMY2A; MGAM; NR5A2; PPAPA; RBP4	Hops
9,10-secocholesta-5Z,7E,10(19)-triene-3b,24,25-triol	AMY2A; HSD11B1; MGAM; NR5A2	Ginger
a-amorphane (cadinane)	RBP4	aniseed
a-amorphene	RBP4	Basil; black pepper; cardamom: clove: lemongrass
a-amyrin	AMY2A	Rosemary; thyme
abietatriene	RBP4	Rosemary
a-bisabolen-12-ol	RBP4	Dill
a-bisabolene	RBP4	Basil; cardamom; ginger; hops; oregano
a-bulnesene	RBP4	Oregano
abyssinone-II	AKR1B1; AMY2A; DPP4; MGAM; NR5A2; PPARA; PTPN9; RBP4; RXRA	Liquorice
acacetin	MGAM; RBP4	Fennel
acacetin 7-O-[4'''-O-acetyl-b-D-apiofuransyl-(1-3)]-b-D-xylopyranoside	AMY2A; DPP4; FBP1; MGAM; NR5A2; PDK2; PPARA; PPARD; PPARG; PTPN9	Oregano

acacetin 7-O-[6'''-O-acetyl-b-D-galactopyranosyl-(1-3)]-b-D-xylopyranoside	AMY2A; DPP4; FBP1; MGAM; NR5A2; PDK2; PPARD; PTPN9; PYGL	Oregano
acacetin-7-O-[6'''-O-acetyl-b-D-galactopyranosyl-(1-2)]-b-D-glucopyranoside	AMY2A; DPP4; HSD11B1; MGAM; NR5A2; PDK2; PTPN9; PYGL	Oregano
acacetin-7-O-rutinoside	AMY2A; DPP4; FBP1; GCK; INSR; MGAM; NR5A2; PDK2; PPARA; PPARD; PTPN9; PYGL	Fennel
a-cadinene	RBP4	Allspice; sage
a-cadinol	RBP4	Allspice; aniseed; basil; clove; oregano; thyme; yarrow
a-calacorene	RBP4	Clove; dill; rosemary; yarrow
achillinin-A	RBP4	Yarrow
achillinin-B	AMY2A; DPP4; FBP1; HSD11B1; MGAM; NR5A2; PDK2	Yarrow
achillinin-C	AMY2A; HSD11B1; MGAM; PDK2; PTPN9	Yarrow
achiterpenoside-A	AMY2A	Yarrow
a-curcumene	RBP4	Black pepper; caraway; cumin; ginger; turmeric; yarrow
adhumulone	MGAM	Hops
adlupulone	MGAM; NR5A2	Hops
a-guaiene	RBP4	Basil; clove; lemongrass; oregano
a-gurjunene	RBP4	Allspice; black pepper; lemongrass; oregano; thyme
a-hydroxyhydrocaffeic acid	MGAM	Basil
alangioside-A	MGAM	Bay leaves

albiziasaponin-B	AMY2A; DPP4; FBP1; MGAM; PDK2; PTPN9; PYGL	Liquorice
allo-aromadendrene	RBP4	Allspice; basil; bay leaves; cardamom; clove; dill; ginger; marjoram; oregano; sage; thyme
alpha-copaene	RBP4	Allspice; basil; bay leaves; black pepper; caraway; cardamom; clove; fennel; ginger; hops; nutmeg; paprika; parsley; rosemary
amburoside-A	AMY2A; DPP4; MGAM; NR5A2; PPARA; RBP4	Oregano
amentoflavone	AKR1B1; AMY2A; DPP4; FBP1; GCK; INSR; MGAM; NR5A2; PDK2; PPARA; PPARD; PPARG; PTPN9; PYGL	Cumin; yarrow
ampelopsin	MGAM; PPARA	Allspice
a-muurolene	RBP4	Aniseed; black pepper; clove; ginger; lemon balm; lemongrass; oregano; paprika; rosemary; sage
anemosapogenin	NR5A2; PTPN9	Bay leaves
anisoxide	RBP4	Star anise
anthemol	MGAM	Caraway
a-oxobisabolene	RBP4	Lemongrass; turmeric
a-patchoulene	RBP4	Dill
a-p-dimethylstyrene	MGAM	Cardamom; nutmeg
apigenin	AMY2A; MGAM; NR5A2; RBP4	Aniseed; basil; black pepper; cumin; dill; fennel; fenugreek; lemon balm;

		lemongrass; marjoram; oregano; parsley; rosemary; sage; thyme; yarrow
apigenin 7-O-[6'''-O-acetyl-b-D-galactopyranosyl-(1-3)]-b-D-xylopyranoside	AMY2A; DPP4; FBP1; MGAM; NR5A2; PDK2; PPARA; PPARD; PTPN9; PYGL	Oregano
apigenin acetylglucoside	AMY2A; DPP4; FBP1; MGAM; NR5A2; PPARA; PTPN9	Sage
apigenin-4-O-glucoside	AMY2A; DPP4; HSD11B1; MGAM; NR5A2; PDK2; PPARA; PTPN9	Yarrow
apigenin-6-C-[B-D-xylopyranosyl-(1'''-2")-B-D-galactopyranoside]-7-O-B-D-(6''''-O-pcoumaroylglucopyranoside)	AMY2A; DPP4; MGAM; PDK2; PPARA; PTPN9	Clove
apigenin-6-C-[B-D-xylopyranosyl-(1'''-2")-B-D-galactopyranoside]-7-O-B-D-glucopyranoside	AMY2A; MGAM; NR5A2; PDK2	Clove
apigenin-7-O-glucoside	AMY2A; DPP4; FBP1; MGAM; NR5A2; PDK2; PTPN9	Aniseed; cumin; fenugreek; lemon balm; marjoram; oregano; rosemary; sage; yarrow
apigenin-7-O-glucuronide	AMY2A; DPP4; FBP1; INSR; MGAM; NR5A2; PDK2; PPARA; PTPN9	Fennel; oregano
apigenin-7-O-rutinoside	AMY2A; DPP4; FBP1; HSD11B1; INSR; MGAM; NR5A2; PDK2; PPARA; PPARG; PTPN9; PYGL	Oregano; rosemary
apiin	AMY2A; DPP4; FBP1; HSD11B1; INSR;	Parsley

	MGAM; NR5A2; PPARA; PPARG; PTPN9	
apioglycyrrhizin	AMY2A; DPP4; FBP1; MGAM; PDK2; PTPN9; PYGL	Liquorice
araboglycyrrhizinic	AMY2A; DPP4; FBP1; INSR; MGAM; PDK2; PTPN9; PYGL	Liquorice
arachidic acid	RBP4	Aniseed; basil; caraway; cardamom; dill; ginger; paprika; turmeric
arjunolic acid	AMY2A	Clove
aromadendrin	PPARA; RBP4	Thyme
ar-turmerol	RBP4	Turmeric
ar-turmerone	RBP4	Turmeric
a-santalene	RBP4	Black pepper; turmeric
a-santalol	RBP4	Marjoram; sage; turmeric
a-selinene	RBP4	Allspice; black pepper; cardamom; clove; hops; lemongrass; oregano; thyme; turmeric; yarrow
asiatic acid	MGAM	Clove; rosemary
asparasaponin I	AMY2A; DPP4; FBP1; MGAM; NR5A2	Fenugreek
a-terpineol-8-O-B-D-(6-O-galloyl)glucopyranoside	MGAM; PDK2; PPARA; PTPN9	Allspice
a-terpinyl[a-L-(2-O-galloyl)arabinofuranosyl]-(1-6)-B-D-glucopyranoside	AMY2A; DPP4; FBP1; MGAM; PPARA; PTPN9	Allspice
a-turmerol	RBP4	Turmeric
a-turmerone	RBP4	Turmeric
augustic acid	AMY2A	Rosemary

auraptene	AKR1B1; MGAM; NR5A2; RBP4	Dill
a-ylangene	RBP4	Bay leaves; black pepper; cardamom; dill; oregano; rosemary
bacilicin	MGAM	Basil
b-amyrin	AMY2A; DPP4; MGAM; NR5A2; PDK2; PTPN9	Liquorice; rosemary; thyme
basilimoside	AMY2A	Basil
b-bisabolene	RBP4	Aniseed; black pepper; caraway; cardamom; clove; dill; ginger; hops; oregano; parsley; thyme; turmeric
b-caryophyllene	RBP4	Allspice; basil; bay leaves; black pepper; caraway; cardamom; cinnamon; clove; cumin; dill; fennel; fenugreek; ginger; hops; lemon balm; liquorice; marjoram; nutmeg; oregano; parsley; rosemary; sage; thyme; turmeric; yarrow
b-copaene	RBP4	Bay leaves; black pepper
b-cubebene	NR5A2; RBP4	Basil; bay leaves; black pepper; cardamom; dill; ginger; lemon balm; nutmeg; rosemary; turmeric
b-curcumene	RBP4	Turmeric
behenic acid	RBP4	Ginger
b-elemol	RBP4	Hops
benzylbenzoate	RBP4	Cinnamon; clove
betulin	NR5A2	Rosemary
betulinic acid	AMY2A; NR5A2; PTPN9	Clove: rosemary; sage
b-germacene	RBP4	Turmeric

b-guaiene	RBP4	Bay leaves; black pepper; clove; hops; rosemary; thyme; yarrow
bicyclo(4.4.0)dec-1-en,2-isopropyl-5-methyl-9-methylene	RBP4	Bay leaves
bicyclo[7.2.0]undecane, 10,10-dimethyl-2,6-bis(methylene)	RBP4	Turmeric
bicyclogermacrene	RBP4	Basil; bay leaves; marjoram; oregano; yarrow
bisacurone B	RBP4	Turmeric
bisacurone C	RBP4	Turmeric
bisdesmethoxycurcumin	AKR1B1; MGAM; NR5A2; RBP4	Turmeric
brachyamide B	AKR1B1; NR5A2; PPARA; RBP4	Black pepper
b-selinene	RBP4	Allspice; basil; bay leaves; black pepper; caraway; cardamom; oregano; yarrow
b-sesquiphellandrene	RBP4	Aniseed; basil; ginger; lemongrass; oregano; turmeric
b-sitostenone	AMY2A; MGAM; NR5A2	Cardamom; thyme
b-sitosterol	AMY2A; DPP4; HSD11B1; MGAM; NR5A2; PPARG	Basil; bay leaves; black pepper; caraway; cardamom; lemon balm; liquorice; saffron; thyme; turmeric
b-sitosterol-3-O-B-D-glucoside	MGAM	Clove
b-turmerone	RBP4	Turmeric
bulnesol	RBP4	Hops
cadalene	RBP4	Cinnamon; rosemary; yarrow
caffeic acid	MGAM	Basil; black pepper; caraway; cinnamon; clove; cumin; dill; fennel; fenugreek; lemon balm; lemongrass; marjoram; oregano; rosemary; saffron; sage; thyme; yarrow

caffeoyl-N-tryptophan	AKR1B1; AMY2A; DPP4; FBP1; MGAM; PDK2; PPAPA; RBP4; RXRA	Dill
calamene	RBP4	Basil; black pepper; clove; oregano; rosemary; thyme
calarene	RBP4	Bay leaves; thyme; yarrow
calebin a	AKR1B1; MGAM; PPARA	Turmeric
campesterol	AMY2A; DPP4; HSD11B1; MGAM; NR5A2; PPARG; PYGL	Bay leaves; caraway; thyme
capsaicin	MGAM	Paprika; allspice
capsanthin-3,6-epoxide	AMY2A; NR5A2	Paprika
capsanthin-5,6-epoxide	AMY2A; NR5A2	Paprika
capsiate	RBP4	Paprika
carbenoxolone	AMY2A; DPP4; FBP2; MGAM; PDK2; PTPN9	Liquorice
carlinoside	AMY2A; DPP4; FBP1; INSR; MGAM; NR5A2; PPARG	Lemongrass
carnosic acid	AMY2A; MGAM; RBP4	Basil; fennel; marjoram; oregano; rosemary; sage; thyme
carnosol	MGAM	Marjoram; oregano; rosemary; sage
carpaine	AMY2A; DPP4; MGAM; NR5A2; PPARD	Fenugreek
caryophyllene alcohol	RBP4	Dill
caryophyllenol II	RBP4	Thyme

cassiatannin a	AMY2A; DPP4; FBP1; MGAM; NR5A2; PDK2; PTPN9; RBP4	Cinnamon
cassioccidentalin B	AMY2A; DPP4; FBP1; HSD11B1; MGAM; NR5A2; PDK2; PPARG; PTPN9; PYGL	Lemongrass
catechin	AMY2A; MGAM; NR5A2; RBP4	Basil; bay leaves; cinnamon; cumin; fenugreek; hops; lemon balm; oregano; rosemary; thyme
catechin gallate	AKR1B1; AMY2A; DPP4; MGAM; NR5A2; PDK2; PPARA; PTPN9; PYGL	Hops
chamazulene	RBP4	Yarrow
chavicine	AKR1B1; NR5A2; PPARA; RBP4; RXRA	Black pepper
chlorogenic acid	MGAM; PTPN9; RBP4	Aniseed; basil; black pepper; caraway; cinnamon; cumin; dill; fennel; fenugreek; lemon balm; lemongrass; marjoram; oregano; rosemary; saffron; sage; thyme; yarrow
chrysanthone c	RBP4	Yarrow
chrysoeriol	AMY2A; RBP4	Parsley
cinnamatannin B1	AMY2A; DPP4; FBP1; MGAM; NR5A2; PDK2; PTPN9; PYGL; RBP4	Cinnamon
cinnamon-3c(Ec)-1Gc(EGc)-tetramer	AMY2A; DPP4; MGAM; PDK2; PYGL	Cinnamon
cinnamon-c(Ec)-tetramer 1	AMY2A; DPP4; FBP1; INSR; MGAM; PDK2	Cinnamon

cinnamon-c(Ec)-tetramer 2	AMY2A; DPP4; MGAM; PDK2; PTPN9	Cinnamon
cinnamophilin	AMY2A; MGAM; NR5A2; RBP4	Cinnamon
cinnamtannin D1	AMY2A; DPP4; FBP1; MGAM; PDK2; PTPN9; PYGL	Cinnamon
cis-11,14-eicosadienoic acid	RBP4	Ginger
cis-g-bisabolene	RBP4	Caraway; clove; ginger; hops; turmeric
cis-miyabenol c	AMY2A; DPP4; MGAM; PPARA; PTPN9; PYGL	Fennel
cis-muurola-3-5-diene	RBP4	Basil
clerosterol	AMY2A; DPP4; HSD11B1; MGAM; NR5A2; PPARG	Caraway
clovane-2,9-diol	RBP4	Clove
cnidilin	RBP4	Parsley
cohumulone	MGAM	Hops
colettiside I	AMY2A; FBP1; MGAM	Fenugreek
colupulone	MGAM; NR5A2	Hops
coniferin	RBP4	Caraway
cosmosiin	AMY2A; DPP4; FBP1; MGAM; NR5A2; PDK2; PPARA; PTPN9; RXRA	Parsley
costunolide	NR5A2; RBP4	Bay leaves
coumaperine	MGAM; RBP4	Black pepper
crisimaritin	AMY2A; FBP1; MGAM; NR5A2; PDK; PPARA; PTPN9; RBP4	Rosemary; sage; thyme

crocin 1	AMY2A; DPP4; FBP1; PDK2	Saffron
crocin 2	MGAM; NR5A2; PDK2; PPARA; PTPN9	Saffron
crocin 3	MGAM; PPARA	Saffron
cryptochlorogenic acid	FBP1; MGAM; PTPN9	Aniseed; cumin; dill; fennel; lemongrass; yarrow
cubebin	AKR1B1; MGAM; NR5A2; PPARA; RBP4	Black pepper
cuminaldehyde	MGAM	Basil; caraway; cardamom; cumin; fennel; fenugreek; liquorice
cuminoid E	PPARA	Cumin
cuminol	MGAM	Caraway; cumin; dill; nutmeg
curculonone_c	RBP4	Turmeric
curculonone_D	RBP4	Turmeric
curcumenol	RBP4	Turmeric
curcumenone	RBP4	Turmeric
curcumin (enol form)	AKR1B1; AMY2A; MGAM	Ginger; turmeric
curcumin (keto form)	AKR1B1; AMY2A; PPARA; RBP4	Turmeric
curcuphenol	RBP4	Turmeric
curdione	RBP4	Turmeric
curlone	RBP4	Turmeric
cyanidin	AKR1B1; AMY2A; DPP4; FBP1; HSD11B1; MGAM; NR5A2; PDK2; PPARA; PTPN9; RBP4	Bay leaves; Nutmeg
cyanidin-3-O-glucoside	AMY2A; FBP1; MGAM; NR5A2; PDK2	Bay leaves

cyanidin-3-O-rutinoside	AMY2A; DPP4; FBP1; HSD11B1; MGAM; NR5A2; PDK; PPARA; PTPN9	Bay leaves
cycloaretnol	AMY2A; GCK; HSD11B1; MGAM; NR5A2; PPARG	Caraway
cyclocurcumin	AKR1B1; AMY2A; MGAM; PPARA	Turmeric
cyclohexadecane	RBP4	Bay leaves
cyclooctacosane	AMY2A; MGAM; NR5A2	Bay leaves
cyclopropa[5,6]-a-nor-5a-androstane-3-dione,3,6B-dihydro-17b-h	NR5A2	Ginger
cyclotetracosane	AMY2A; HSD11B1; MGAM; NR5A2; PPARD; PPARG	Bay leaves
cymbopogonol	AKR1B1; AMY2A; HSD11B1; MGAM; NR5A2; PTPN9; PYGL	Lemongrass
cynaroside	AMY2A; DPP4; FBP1; MGAM; NR5A2; PPARA; PTPN9	Lemongrass
daidzein	AMY2A; NR5A2; RBP4	Dill; hops
danshensu	MGAM	Sage
d-cadinene	RBP4	Allspice; basil; bay leaves; black pepper; caraway; cardamom; cinnamon; clove; dill; fennel; ginger; hops; lemongrass; oregano; rosemary; thyme; yarrow
deca-2E,4E,6E,8Z-tetraenoic acid piperideide	RBP4	Yarrow
deca-2E,4E,6Z,8Z tetraenoic acid piperideide	RBP4	Yarrow
deca-2E,4E,6Z-trienoic acid piperideide	RBP4	Yarrow

deca-2E,4E,8Z-trienoic acid isobutylamide	RBP4	Yarrow
deca-2E,4E,8Z-trienoic acid piperideide	RBP4	Yarrow
deca-2E,4E,8Z-trienoic acid piperidide	RBP4	Yarrow
deca-2E,4E-dienoic acid piperidide	RBP4	Yarrow
deca-2E,4E-dienoic acid tyramide	MGAM; RBP4	Yarrow
dehydroaromadendrene	RBP4	Basil
dehydrocostunolide	NR5A2; RBP4	Bay leaves
dehydrocostus lactone	RBP4	Bay leaves
dehydrocurdione	RBP4	Turmeric
dehydrodieugenol	MGAM	Clove
dehydropipernonaline	AKR1B1; AMY2A; FBP1; GCK; MGAM; NR5A2; PPARA; RBP4; RXRA	Black pepper
dehydroretrofractamide C	RBP4	Black pepper
delta-5-avenasterol (Isofucosterol)	AMY2A; HSD11B1; MGAM; NR5A2	Caraway
delta-7-avenasterol	AMY2A; DPP4; HSD11B1; MGAM; NR5A2; PDK2; PPARG	Caraway
demethoxy-6-gingerol	RBP4	Ginger
demethoxy-6-shogaol	RBP4	Ginger
desacetylmatricarin	RBP4	Yarrow
desmethoxycurcumin	AKR1B1; AMY2A; MGAM; NR5A2; RBP4	Turmeric
desmethylxanthohumol	MGAM; PPARA; RBP4; RXRA	Hops
diacetoxy-6-gingerdiol	RBP4	Ginger
dicumylperoxide	RBP4	Turmeric

didymin	AMY2A; DPP4; FBP1; MGAM; NR5A2; PDK2; PPARA; PTPN9	Oregano
di-epi-cedrene	NR5A2	Aniseed; clove; cumin; turmeric
dihydro-ar-turmerone	RBP4	Turmeric
dihydroguaiaretic acid	MGAM	Mace
dihydrokaempferol-7-O-B-D-glucopyranoside	AMY2A; DPP4; FBP1; MGAM; PTPN9	Saffron
dihydropipericide	PPARA	Black pepper
dihydroxyxanthohumol	AKR1B1; MGAM	Hops
dioscin	AMY2A; DPP4; FBP1; MGAM; NR5A2; PDK2; PTPN9	Fenugreek
diosgenin	AMY2A; HSD11B1; MGAM; NR5A2	Fenugreek
diosmetin	MGAM; RBP4	Yarrow
diosmetin-7-O-B-D-glucopyranoside	AMY2A; DPP4; FBP1; MGAM; PTPN9	Parsley
echinatin	MGAM; RBP4	Liquorice
eicosapentaenoic acid	RBP4	Ginger
ellagic acid	PPARA; PTPN9; RBP4	Aniseed; clove; dill; lemon balm; sage
emodin	AMY2A; PPARA; RBP4	Liquorice
epicatechin	AMY2A; MGAM; NR5A2; RBP4	Cinnamon; cumin; dill; hops; lemon balm; nutmeg; oregano; rosemary; sage; thyme
epicatechin gallate	AMY2A; DPP4; INSR; MGAM; NR5A2; PPARA; PYGL	Aniseed; hops
epigallocatechin	AMY2A; DPP4; FBP1; MGAM; NR5A2; PDK1; PPARG; PTPN9	Lemon balm; oregano; thyme

epigallocatechin gallate	AMY2A; DPP4; FBP1; MGAM; NR5A2; PDK1; PPARG; PTPN9	Sage
epiisorosmanol	MGAM	Rosemary
epiprocurcumenol	RBP4	Turmeric
epirosmanol	DPP4; MGAM	Rosemary; sage
epirosmanol ester of 12-O-methylcarnosic acid	AMY2A	Sage
eremanthin	NR5A2; RBP4	Bay leaves
eremophilene	RBP4	Bay leaves
eriocitrin	AMY2A; DPP4; FBP1; HSD11B1; MGAM; NR5A2; PDK2; PPARA; PPARD; PPARG; PTPN9; PYGL	Fennel; sage
eriodictyol	MGAM; PPARA; RBP4	Oregano; rosemary; thyme
eriodictyol-7-O-glucoside	AMY2A; FBP1; MGAM; PPARA; PTPN9	Lemon balm
estra-1,3,5(10)-trien-17b-ol	AMY2A; MGAM; NR5A2; RBP4	Ginger
ethyl iso-allocholate	MGAM	Lemon balm
euchrenone A5	AKR1B1; AMY2A; DPP4; HSD11B1; MGAM; NR5A2; PDK2; PPARA; PPARD; PPARG; PYGL	Liquorice
eudesmol acetate	RBP4	Bay leaves
eugeniin	AKR1B1; AMY2A; DPP4; FBP1; INSR; MGAM; PDK2; PPARA; PTPN9; PYGL	Clove

eugenol-4-O-B-D-(6-O-galloyl)glucopyranoside	MGAM; NR5A2; PDK2; PPARA; PTPN9	Allspice
fenretinide	MGAM; PPARA	Ginger
fenugreekine	DPP4; FBP1; HSD11B1; MGAM; NR5A2; PDK2; PPARA; PPARG; PTPN9; PYGL	Fenugreek
fenugrin A	AKR1B1; RBP4; RXRA	Fenugreek
ferruginol	AMY2A; MGAM; RBP4	Sage
flavone	AMY2A; MGAM; NR5A2; PPARA; RBP4; RXRA	Cumin
foeniculoside I	AMY2A; DPP4; FBP1; MGAM; NR5A2; PDK2; PPARD; PTPN9; PYGL	Fennel
foeniculoside II	MGAM; PDK2	Fennel
foeniculoside III	AMY2A; DPP4; MGAM; PDK2; PPARA	Fennel
foeniculoside IV	DPP4; MGAM	Fennel
foeniculoside VI	MGAM	Fennel
foeniculoside VIII	MGAM	Fennel
foeniculoside X	MGAM; NR5A2	Fennel
foeniculoside XI	AMY2A; DPP4; FBP1; MGAM; PDK2; PTPN9; PYGL	Fennel
folic acid	AMY2A; DPP4; FBP1; MGAM; PDK2; PPARA; PTPN9; RBP4	Fenugreek; ginger
formononetin	RBP4	Liquorice
galbacin	AMY2A; MGAM; NR5A2; PDK2; PPARA	Nutmeg

gallocatechin	MGAM	Rosemary
gancaonin L	AMY2A; DPP4; HSD11B1; MGAM; RBP4	Liquorice
gardenin B	RBP4	Thyme
gazaniolide	NR5A2; RBP4	Bay leaves
g-cadinene	RBP4	Allspice; basil; bay leaves; cardamom; clove; dill; lemongrass; oregano; rosemary; sage; thyme; yarrow
gemin D	AMY2A; DPP4; MGAM; PDK2; PTPN9; PYGL	Clove
genistein	AMY2A; PPARA; RBP4	Liquorice
genkwanin	AMY2A; MGAM; NR5A2; RBP4	Liquorice; rosemary; sage; thyme
geraniyllinalool	RBP4	Lemon balm; turmeric
germacra-4(15),5,10(14)-trien-1a-ol	RBP4	Clove
germacrene A	RBP4	Basil; bay leaves
germacrene B	RBP4	Black pepper; caraway; thyme
germacrene D	NR5A2	Allspice; aniseed; basil; bay leaves; black pepper; caraway; cardamom; clove; cumin; dill; ginger; lemon balm; lemongrass; nutmeg; oregano; parsley; rosemary; thyme; turmeric; yarrow
germacrone	PPARA; RBP4	Lemon balm; turmeric
g-gurjunen epoxide	RBP4	Turmeric
g-gurjunene	RBP4	Bay leaves; black pepper
g-himachalene	RBP4	Aniseed; dill; turmeric
giganteone A	AKR1B1; AMY2A; MGAM; NR5A2; PDK2; PPARA	Nutmeg

giganteone C	AKR1B1; MGAM; NR5A2; PPARA	Nutmeg
gitogenin	AMY2A; DPP4; HSD11B1; MGAM; NR5A2	Fenugreek
gitoxigenin	AMY2A; MGAM; NR5A2; PDK2; PTPN9	Turmeric
glabranin	AMY2A; MGAM; NR5A2; PPARA; PTPN9; RBP4	Liquorice
glabrene	AMY2A; DPP4; MGAM; NR5A2; PDK2; PPARA; PTPN9; RBP4	Liquorice
glabric acid	AMY2A; DPP4; FBP1; MGAM; NR5A2; PDK2; PTPN9; PYGL	Liquorice
glabridin	AKR1B1; AMY2A; DPP4; HSD11B1; MGAM; NR5A2; PDK2	Liquorice
glabrocoumarin	AKR1B1; AMY2A; DPP4; HSD11B1; MGAM; NR5A2; PDK2; PPARA; PTPN9; PYGL; RBP4	Liquorice
glabrol	AMY2A; DPP4; INSR; MGAM; NR5A2; PDK2; PPARA; PPARG; RBP4; RXRA	Liquorice
glabrolide	AMY2A; DPP4; FBP1; MGAM; NR5A2; PDK2; PTPN9; PYGL	Liquorice

glabrone	AMY2A; DPP4; HSD11B1; MGAM; NR5A2; PDK2; PPARA	Liquorice
glicoricone	MGAM	Liquorice
glisoflavone	AMY2A; DPP4; MGAM	Liquorice
glucoliquiritin apioside	AMY2A; DPP4; FBP1; INSR; MGAM; NR5A2; PDK2; PTPN9	Liquorice
glyasperin C	RBP4	Liquorice
glyasperin D	PPARA	Liquorice
glyasperin F	AMY2A; DPP4; MGAM; NR5A2; PPARA; PTPN9; RBP4	Liquorice
glycerol-1-O-A-D-glucuronide-3-O-benzoyl-ester	MGAM	Cumin
glycycoumarin	AKR1B1; AMY2A; MGAM; PPARA; PTPN9; RBP4	Liquorice
glycycumarin	AKR1B1; PPARA; RBP4	Liquorice
glycyrin	PPARA	Liquorice
glycyrol	AMY2A; MGAM; NR5A2; PPARD; PTPN9	Liquorice
glycyroside	FBP1; MGAM; NR5A2; PYGL	Liquorice
glycyrrhetic acid	AMY2A; FBP1; MGAM; PTPN9; PYGL	Liquorice
glycyrrhetinic acid	AMY2A; DPP4; MGAM; NR5A2; PTPN9	Liquorice

glycyrrhetol	AMY2A; DPP4; FBP1; HSD11B1; MGAM; NR5A2	Liquorice
glycyrrhisoflavon	AMY2A; DPP4; HSD11B1; MGAM; PPARA; PYGL; RBP4	Liquorice
glycyrrhizin	AMY2A; DPP4; FBP1; MGAM; PDK2; PTPN9; PYGL	Liquorice
glyinflanin G	AMY2A; DPP4; FBP1; FFAR1; HSD11B1; INSR; MGAM; NR5A2; PDK2; PPARA; PPARD; PPARG; PTPN9; PYGL	Liquorice
glyinflanin H	AMY2A; DPP4; MGAM; NR5A2; RBP4	Liquorice
glyzaglabrin	AMY2A; MGAM; NR5A2; RBP4	Liquorice
g-muurolene	RBP4	Allspice; basil; black pepper; cardamom; clove; ginger; lemongrass; oregano; thyme; yarrow
graveolone	PPARA; RBP4	parsley
g-sitosterol	NR5A2	Cardamom
g-terpinene	MGAM	Allspice; basil; bay leaves; black pepper; caraway; cardamom; clove; cumin; dill; fennel; ginger; hops; marjoram; nutmeg; oregano; parsley; rosemary; sage; star anise; thyme; turmeric; yarrow
g-tocopherol	PPARA	Cardamom; ginger; paprika
guineensine	PPARA	Black pepper

harman	RBP4	Saffron
hesperetin	AMY2A; DPP4; FBP1; MGAM; NR5A2; PDK2; PPARA; PTPN9; PYGL	Cumin; dill; lemon balm; rosemary
hesperidin	AMY2A; DPP4; FBP1; MGAM; NR5A2; PDK2; PPARA; PPARD; PPARG; PTPN9; PYGL	Dill; fennel; lemon balm; marjoram; rosemary; thyme
himachalene epoxide	RBP4	Liquorice
hispaglabridin A	AMY2A; DPP4; HSD11B1; MGAM; NR5A2; PDK2; PPARA; PPARG; PTPN9	Liquorice
hispaglabridin B	AKR1B1; AMY2A; DPP4; FBP1; FFAR1; HSD11B1; INSR; MGAM; NR5A2; PDK2; PPARA; PPARD; PPARG	Liquorice
hispidulin	MGAM; RBP4	Rosemary; sage; thyme
hispidulin glucuronide	AMY2A; MGAM; PDK2; PTPN9	Sage
hispidulin rutinoside	AMY2A; DPP4; FBP1; MGAM; MR5A2; PTPN9	Rosemary
homocapsaicin	MGAM	Paprika
homoplantaginin	AMY2A; MGAM; PDK2; PTPN9	Rosemary; sage
hop-17(21)-en-3b-ol	AKR1B1; AMY2A; HSD11B1; MGAM;	Hops

	NR5A2; PDK2; PPARD; PTPN9	
hop-17(21)-en-3b-yl acetate	AMY2A; MGAM; NR5A2; PDK2; PPARD	Hops
hopenone I	AKR1B1; AMY2A; FBP1; HSD11B1; MGAM; NR5A2; PPARD; PTPN9; PYGL	Hops
humulone	MGAM; NR5A2	Hops
humulusol	AMY2A; DPP4; MGAM; PPARA; PPARD; PPARG	Hops
hyperoside	MGAM; NR5A2	Allspice; black pepper; fennel; star anise
icariside D1	FBP1; MGAM; PTPN9	Aniseed
icariside D2	RBP4	Aniseed
illicinole	RBP4	Star anise
imperatorin	AMY2A; MGAM; NR5A2; PPARA; RBP4	Parsley
isoangustone A	AMY2A; DPP4; FBP1; HSD11B1; MGAM; NR5A2; PDK2; PPARA; PPARG; PTPN9	Liquorice
isocaryophyllene	RBP4	Black pepper; dill; thyme
isochavicine	MGAM; NR5A2; PPARA; RBP4; RXRA	Black pepper
isoglabrolide	AMY2A; DPP4; FBP1; HSD11B1; MGAM; NR5A2; PDK2; PPARD; PTPN9; PYGL	Liquorice
isoglycyrol	AMY2A; DPP4; FBP1; HSD11B1; MGAM;	Liquorice

	NR5A2; PPARA; PPARD; PPARG; PYGL	
isoimperatorin	AMY2A; MGAM; NR5A2; RBP4	parsley
isolariciresinol-3a-O-B-D-glucopyranoside	MGAM	Sage
isoliquiritigenin	MGAM; RBP4	Liquorice
isoliquiritin	AMY2A; DPP4; MGAM; PPARA	Liquorice
isoliquiritin apioside	AMY2A; DPP4; FBP1; MGAM; NR5A2; PTPN9	Liquorice
isolongifolene-4,5,9,10-dehydro	RBP4	Lemongrass
isoorientin	AMY2A; DPP4; FBP1; MGAM	Aniseed; fenugreek; lemongrass; yarrow
isoorientin 2 -O-rhamnoside	AMY2A; DPP4; FBP1; MGAM; PTPN9	Lemongrass
isopaulitin	NR5A2; RBP4	Yarrow
isopiperine	AKR1B1; NR5A2; PPARA; RBP4; RXRA	Black pepper
isopiperolein B	AKR1B1; NR5A2; PPARA; RBP4	Black pepper
isoprocurcumenol	RBP4	Turmeric
isopulegol	MGAM; NR5A2; RBP4	Hops; rosemary
isorhamnetin	AMY2A; MGAM; RBP4	Caraway; cinnamon; fennel; parsley; rosemary
isorhamnetin-3-O-galactoside	MGAM; PPARA	Fennel
isorhamnetin-3-O-glucoside	MGAM; NR5A2; PTPN9	Fennel; rosemary
isorhamnetin-3-O-glucuronide	MGAM; PTPN9; PYGL	Fennel; dill

isorhamnetin-3-O-rutinoside	AMY2; DPP4; FBP1; MGAM; PDK2; PPARD; PTPN9; PYGL	Black pepper; fennel
isorosmanol	MGAM	Rosemary
isosakuranetin	PPARA	Rosemary
isoschaftoside	AMY2A; FBP1; MGAM; PPARA; PYGL	Lemongrass; liquorice
isoscoparin	AMY2A; FBP1; PPARG	Lemongrass
isoviolanthin	AMY2A; DPP4; MGAM; PPARA; PYGL	Liquorice
isovitexin	AMY2A; FBP1; MGAM	aniseed; fenugreek; liquorice
isoxanthohumol	AMY2A; MGAM; NR5A2; PPARA; RBP4	hops
ispaglabridin B	AMY2A; DPP4; FBP1; FFAR1; HSD11B1; INSR; MGAM; NR5A2; PPARA; PPARD; PPARG	Liquorice
kaempferide-3-O-glucuronide	FBP1; MGAM	Oregano
kaempferol	MGAM; PPARA; RBP4	Bay leaves; black pepper; cinnamon; clove; dill; fennel; fenugreek; hops; lemon balm; lemongrass; oregano; rosemary; saffron; star anise; thyme
kaempferol-3,7,4'-tri-O-B-glucopyranoside	AMY2A; DPP4; FBP1; MGAM; NR5A2; PTPN9	Saffron
kaempferol-3,7-dirhamnopyranoside	AMY2A; DPP4; FBP1; HSD11B1; INSR; MGAM; NR5A2; PPARA; PPARD; PTPN9	Bay leaves

kaempferol-3-O-a-L-(2"-E-p-coumaroyl)rhamnoside	AKR1B1; AMY2A; DPP4; FBP1; GCK; HSD11B1; INSR; MGAM; NR5A2; PDK2; PPARA; PPARD; PPARG; PTPN9	Bay leaves
kaempferol-3-O-a-L-(2-O-B-D-glucopyranosyl)rhamnoperanoside-7-O-B-D-(6-O-acetyl)glucopyranoside	AMY2A; DPP4; HSD11B1; MGAM; NR5A2	Saffron
kaempferol-3-O-a-L-(2-O-B-D-glucopyranosyl)rhamnopyranoside-7-O-B-D-glucopyranoside	AMY2A; HSD11B1; MGAM; NR5A2; PPARA	Saffron
kaempferol-3-O-a-L-(3",4"-di-(E)-p-coumaroyl)rhamnoside	AKR1B1; AMY2A; DPP4; FBP1; GCK; HSD11B1; INSR; MGAM; NR5A2; PDK2; PPARA; PPARD; PPARG; PTPN9; PYGL	Bay leaves
kaempferol-3-O-alpha-L-(2",3"-di-E-P-coumaroyl)-rhamnoside	AMY2A; DPP4; GCK; MGAM; NR5A2; PDK2; PPARA; PPARD; PTPN9; PYGL	Fennel
kaempferol-3-O-arabinopyranoside	MGAM	Bay leaves
kaempferol-3-O-arabinoside-7-rhamnoside	AMY2A; DPP4; HSD11B1; MGAM; NR5A2; PPARA; PPARD	Black pepper
kaempferol-3-O-B-D-(2-O-B-D-acetylglucosyl)glucopyranoside	NR5A2	Saffron
kaempferol-3-O-B-D-glucopyranosyl-(1-2)-O-B-D-glucopyranoside-7-O-B-D-glucopyranoside	AMY2A; DPP4; MGAM	Saffron
kaempferol-3-O-galactoside	MGAM	Star anise

kaempferol-3-O-glucoside (astralagin)	MGAM; PTPN9; PYGL	Bay leaves; black pepper; caraway; cumin; dill; fennel; parsley; saffron; star anise;
kaempferol-3-O-glucuronide	MGAM; NR5A2	Dill; fennel
kaempferol-3-O-rhamnoside	MGAM; NR5A2	Bay leaves
kaempferol-3-O-rutinoside (nictoflorin)	AMY2A; DPP4; FBP1; HSD11B1; MGAM; NR5A2; PDK2; PPARD; PTPN9; PYGL	Cumin; fennel; star anise
kaempferol-3-O-sophoricoside-7-O-B-D-glucopyranoside	AMY2A; FBP1; MGAM; NR5A2; PPARA	Saffron
kaempferol-7-O-B-D-glucopyranoside (populnin)	AMY2A; FBP1; MGAM; PDK2; PPARA; PTPN9	Saffron
kaempferol-7-O-B-D-sophoroside	AMY2A; DPP4; FBP1; HSD11B1; MGAM; NR5A2; PTPN9	Saffron
kanzonol H	AMY2A; DPP4; HSD11B1; MGAM; NR5A2	Liquorice
kanzonol U	AMY2A; MGAM; NR5A2; PDK2; PTPN9; RBP4	Liquorice
kanzonol V	AMY2A; DPP4; FBP1; HSD11B1; INSR; MGAM; PPARA; PPARD; PPARG; PTPN9; PYGL	Liquorice

kanzonol W	AMY2A; DPP4; FBP1; HSD11B1; MGAM; NR5A2; PDK2	Liquorice
kanzonol X	AMY2A; DPP4; HSD11B1; MGAM; NR5A2; PDK2; PPARA; PPARG; PTPN9	Liquorice
kanzonol Y	AMY2A; DPP4; MGAM; NR5A2; PPARA	Liquorice
kumatakenin	AMY2A	Liquorice
kurilensin A	AMY2A; MGAM; NR5A2; PYGL	Lemongrass
ladanein	AMY2A; FBP1; MGAM; NR5A2; PDK2; PPARA; PTPN9; RBP4	Rosemary
lauroside C	MGAM	Bay leaves
ledol	RBP4	Caraway; clove
licarin B	AMY2A; DPP4; MGAM; NR5A2; PDK2; PYGL	Nutmeg
licoagrocarpin	AKR1B1; AMY2A; FBP1; MGAM; NR5A2; PPARA; PTPN9	Liquorice
licochalcone A	RBP4	Liquorice
licochalcone B	RBP4	Liquorice
licocoumarin A	AMY2A; DPP4; HSD11B1; MGAM; NR5A2; PPARA; PPARG; PTPN9	Liquorice
licocoumarone	MGAM	Liquorice

licoflavanone A	AMY2A; DPP4; MGAMNR5A2; PDK2; PPARA	Liquorice
licoflavone B	AMY2A; DPP4; FBP1; HSD11B1; INSR; MGAM; NR5A2; PPARA; PPARG; PYGL	Liquorice
licoflavonol	AMY2A; DPP4; MGAM; PPARA	Liquorice
licoisoflavanon	AMY2A; DPP4; HSD11B1; MGAM; NR5A2	Liquorice
licoisoflavone A	AMY2A; MGAM; NR5A2; PPARA	Liquorice
licoisoflavone B	AMY2A; DPP4; FBP1; HSD11B1; MGAM; NR5A2; PPARA	Liquorice
licoricidin	AMY2A; DPP4; MGAM; NR5A2; PPARA; PPARG	Liquorice
licoricone	AMY2A; MGAM; NR5A2; PPARA	Liquorice
licoriphenone	AKR1B1; MGAM; PPARA	Liquorice
licuraside	AMY2A; FBP1; MGAM; PTPN9	Liquorice
licuroside	AMY2A; FBP1; MGAM; NR5A2; PTPN9	Liquorice
lilyn	AMY2A; FBP1; MGAM	Fenugreek

limonin	AMY2A; MGAM; PTPN9	Clove
linderol	AMY2A; GCK; MGAM; NR5A2; PPARA; PPARD; PTPN9	Caraway
linolenic acid	RBP4	Aniseed; basil; black pepper; caraway; cardamom; cinnamon; cumin; dill; fenugreek; ginger; lemon balm; nutmeg; paprika; turmeric
liqcoumarin	RBP4	Liquorice
liquiritigenin	AMY2A; NR5A2; PPARA; RBP4	Liquorice
liquiritin	AMY2A; FBP1; HSD11B1; MGAM; NR5A2; PDK2; PPARA; PPARG	Liquorice
liquiritin apioside	AMY2A; DPP4; HSD11B1; MGAM; NR5A2; PYGL	Liquorice
liquoric acid	AMY2A; DPP4; FBP1; MGAM; PTPN9; PYGL	Liquorice
liquorice glycoside A	AMY2A; DPP4; FBP1; MGAM; NR5A2; PDK2; PPARA; PPARD; PPARG; PTPN9	Liquorice
liquorice glycoside B	AMY2A; DPP4; MGAM; NR5A2; PDK2; PPARD; PPARG; PTPN9	Liquorice
liquorice glycoside C1	AMY2A; DPP4; FBP1; MGAM; NR5A2; PDK2;	Liquorice

	PPARA; PPARD; PTPN9; PYGL	
liquorice glycoside C2	AMY2A; DPP4; FBP1; INSR; MGAM; NR5A2; PDK2; PPARA; PPARD; PPARG; PTPN9; PYGL	Liquorice
liquorice glycoside D1	AMY2A; DPP4; GCK; MGAM; NR5A2; PDK2; PPARA; PPARD; PPARG; PTPN9; PYGL	Liquorice
liquorice glycoside D2	AKR1B1; AMY2A; DPP4; FBP1; GCK; HSD11B1; MGAM; NR5A2; PDK2; PPARA; PPARD; PPARG; PTPN9; PYGL	Liquorice
liquorice saponin A3	AMY2A; DPP4; FBP1; MGAM; PTPN9	Liquorice
liquorice saponin B2	AMY2A; DPP4; FBP1; INSR; MGAM; PDK2; PTPN9; PYGL	Liquorice
liquorice saponin C2	AMY2A; DPP4; FBP1; INSR; MGAM; PPARA; PTPN9; PYGL	Liquorice
liquorice saponin D3	AMY2A; DPP4; FBP1; INSR; MGAM; PDK2; PTPN9; PYGL	Liquorice
liquorice saponin E2	AMY2A; DPP4; FBP1; INSR; MGAM; NR5A2; PDK2; PTPN9; PYGL	Liquorice

liquorice saponin F3 (rha)	AMY2A; DPP4; FBP1; MGAM; NR5A2; PDK2; PPARA; PTPN9	Liquorice
liquorice saponin G2	AMY2A; DPP4; FBP1; MGAM; PDK2; PTPN9	Liquorice
liquorice saponin H2	AMY2A; DPP4; FBP1; MGAM; PTPN9; PYGL	Liquorice
liquorice saponin J2	AMY2A; DPP4; FBP1; MGAM; PDK2; PTPN9	Liquorice
liquorice saponin K2	AMY2A; DPP4; FBP1; MGAM; PDK2; PTPN9; PYGL	Liquorice
liquorice saponin L3 (Rha)	AMY2A; DPP4; FBP1; MGAM; NR5A2; PDK2; PTPN9	Liquorice
lithospermic acid A	AMY2A; DPP4; GCK; HSD11B1; MAGM; NR5A2; PDK2; PYGL	Basil; lemon balm; marjoram; oregano; thyme
lupeol	AMY2A; NR5A2	Lemon balm; sage
lupulone	MGAM; NR5A2	Hops
lupulone derivative 10	MGAM; NR5A2	Hops
lupulone derivative 9	AMY2A; MGAM; NR5A2	Hops
luteolin	AMY2A; MGAM; NR5A2; RBP4	Cumin; dill; fenugreek; hops; lemon balm; lemongrass; oregano; parsley; rosemary; sage; thyme; yarrow
luteolin 2 -O-deoxyosyl-6-C-(6-deoxy-pento-hexos-ulosyl)	AMY2A; DPP4; FBP1; MGAM; NR5A2; PPARG; PTPN9; PYGL	Lemongrass
luteolin-3'-O-(3''-O-acetyl)-B-D-glucuronide	AMY2A; FBP1; HSD11B1; MGAM;	rosemary

	NR5A2; PPARA; PTPN9	
luteolin-3-O-B-D-glucuronide	AMY2A; DPP4; FBP1; HSD11B1; MGAM; NR5A2; PDK2; PPARA; PPARG; PTPN9	Lemon balm; rosemary; sage
luteolin-4-O-glucoside	AMY2A; DPP4; FBP1; INSR; MGAM; PPARG	Yarrow
luteolin-6,8-di-C-glucoside (lucenin-2)	FBP1; MGAM	Marjoram
luteolin-6-C-glucoside	AMY2A; MGAM; NR5A2; PDK2; PPARG	Aniseed; lemongrass
luteolin-7-B-glucuronide	AMY2A; DPP4; FBP1; INSR; MGAM; NR5A2; PDK2; PPARA; PPARG; PTPN9	Lemon balm; oregano; rosemary; sage; yarrow
luteolin-7-O-b-D-glucopyranoside-3'-O-b-D-glucuronopyranoside	AMY2A; DPP4; FBP1; HSD11B1; MGAM; NR5A2; PDK2; PTPN9	Lemon balm
luteolin-7-O-b-D-glucuronopyranoside	AMY2A; DPP4; FBP1; INSR; MGAM; NR5A2; PDK2; PPARA; PTPN9	Aniseed; cumin; fenugreek; lemon balm; lemongrass; marjoram; oregano; rosemary; sage; thyme; yarrow
luteolin-7-O-neohesperidoside	AMY2A; DPP4; FBP1; HSD11B1; INSR; MGAM; NR5A2; PDK2; PPARG; PTPN9; PYGL	Lemongrass
luteolin-7-O-rutinoside	AMY2A; DPP4; FBP1; HSD11B1; MGAM; NR5A2; PDK2; PPARA; PPARD; PPARG; PTPN9; PYGL	Fennel; marjoram; rosemary; sage; thyme

luteolin-diglucuronide	AMY2A; DPP4; FBP1; HSD11B1; INSR; MGAM; NR5A2; PDK2; PPARA; PPARD; PPARG; PTPN9; PYGL	Sage; thyme
lyoniside	AMY2A; MGAM	Bay leaves
macedonoside A	AMY2A; DPP4; FBP1; MGAM; NR5A2; PDK2; PTPN9; PYGL	Liquorice
macedonoside B	AMY2A; DPP4; FBP1; INSR; MGAM; PTPN9; PYGL	Liquorice
macedonoside C	AMY2A; DPP4; FBP1; INSR; MGAM; NRA52; PDK2; PPARA; PTPN9; PYGL	Liquorice
malabaricone B	PPARA; RBP4	Nutmeg
malabaricone C	PPARA; RBP4	Nutmeg
malonylapiin	AMY2A; FBP1; HSD11B1; INSR; MGAM; NR5A2; PPARA; PTPN9; PYGL	Parsley
mangicrocin	AMY2A; DPP4; MGAM; NR5A2; PPARA; PTPN9	Saffron
manool	RBP4	Sage
martynoside	AMY2A; MGAM; NR5A2; PDK2; PPARA	Sage
m-coumaric acid	MGAM	Fenugreek; lemon balm
medioresinol	AMY2A	Cinnamon; rosemary

melitric acid A	AMY2A; DPP4; MGAM; NR5A2; PDK2; PPARA; PPARG	Lemon balm
melitric acid B	AMY2A; GCK; HSD11B1; MGAM; NR5A2; PDK2; PPARA; PPARD; PPARG; PTPN9; PYGL	Lemon balm
methyl achimillate B	RBP4	Yarrow
methyl achimillate C	RBP4	Yarrow
methyl carnosate	MGAM	Rosemary; sage
methyl rosmarinate	AKR1B1; MGMA; PPARA; RBP4	Marjoram; rosemary; thyme
micromeric acid	AMY2A; MGAM	Rosemary
miltirone	MGAM; NR5A2; RBP4	Rosemary
morachalcone A	AKR1B1; AMY2A; MGAM; RBP4; RXRA	Liquorice
myricetin	MGAM; PPARA; RBP4	Allspice; clove; dill; fenugreek; lemon balm; lemongrass; rosemary
myricetin-3-O-galactoside	AMY2A; MGAM; PTPN9	Allspice
myricetin-3-O-glucoside	MGAM; NR5A2	Oregano
n-(2,4-dinitrophenyl)-N-13-(2,6,6-trimethyl-cyclohex-1-enyl)propylider	MGAM; RBP4	Ginger
naphthalene,1,2,3,4,4a,5,6,8a-octahydro-4a,8-dimethyl-2-(1-methylethylidene) (Eudesma-3,7(11)-diene)	RBP4	Ginger; turmeric
naringenin	AMY2A; MGAM; RBP4	Dill; fennel; fenugreek; lemon balm; oregano; rosemary; thyme
naringenin-7-O-glucoside (Prunin)	AMY2A; DPP4; FBP1; MGAM; NR5A2; PTPN9	Fenugreek; liquorice

naringin	AMY2A; DPP4; HSD11B1; MGAM; PDK2; PYGL	Aniseed; lemon balm; thyme
narirutin	AMY2A; DPP4; FBP1; HSD11B1; MGAM; NR5A2; PDK2; PPARD; PPARG; PTPN9; PYGL	Fennel; fenugreek
nectandrin B	MGAM	Nutmeg
neochlorogenic acid	MGAM	Aniseed; caraway; cumin; dill; fennel; lemongrass; oregano; thyme; yarrow
neoisoliquiritin	AKR1B1; AMY2A; FBP1; MGAM; PPARA; PTPN9	Liquorice
neolicuroside	AMY2A; DPP4; FBP1; MGAM; NR5A2; PTPN9; PYGL	Liquorice
neoliquiritin	AMY2A; DPP4; FBP1; MGAM; PTPN9	Liquorice
neophytadiene	AKR1B1; AMY2A; FFAR1; MGAM; NR5A2; PPARA; PPARD; RBP4	Aniseed; bay leaves; dill; thyme
neotigogenin	AMY2A; DPP4; HSD11B1; NR5A2	Fenugreek
nepitrin	AMY2A; MGAM; PPARA; PTPN9	Rosemary
nerolidyl propionate	RBP4	Turmeric
N-feruloyltyramine	MGAM; RBP4	Black pepper
nootkatin	RBP4	Clove
oleanolic_acid	AMY2A; FBP1; HSD11B1;	Clove; fennel; lemon balm; rosemary; sage

	MGAMNR5A2; PDK2; PTPN9	
ombutin-3-O-B-D-glucopyranoside	AMY2A; MGAM	Clove
O-methylshinpterocarpin	AMY2A; MGAM; NR5A2; PDK2; PPARA; PPARD; PPARG	Liquorice
ononin	AMY2A; FBP1; MGAM; PPARA	Liquorice
orientin	AMY2A; INSR; NR5A2; PPARA; PTPN9	Fenugreek; lemongrass
origanoside	PPARA	Oregano
osthol	RBP4	Dill
oxypeucedanin	RBP4	Dill; parsley
oxypeucedanin hydrate	NR5A2; RBP4	Dill; parsley
paulitin	NR5A2; RBP4	Yarrow
p-coumaric-acid-4-glucoside	MGAM	Parsley
p-coumaric-acid-4-O-(2'-O-B-D-apiofuranosyl)-B-D-glucopyranoside	MGAM	Sage
p-cymen-7-ol	MGAM	Bay leaves; oregano; thyme
p-cymen-9-ol	MGAM	Dill; rosemary
p-Cymene	MGAM	Allspice; basil; black pepper; caraway; cardamom; cinnamon; clove; cumin; dill; fennel; hops; marjoram; nutmeg; oregano; parsley; rosemary; sage; star anise; thyme; turmeric; yarrow
p-cymenene	MGAM	Rosemary
pectolinarigenin	AKR1B1; AMY2A; DPP4; FBP1; HSAD11B1; MGAM; NR5A2; PPARA; RBP4	Rosemary
peonidin-3-O-glucoside	MGAM	Bay leaves

peonidin-3-O-rutinoside	AMY2A; DPP4; FBP1; MGAM; PDK2; PPARA; PTPN9; PYGL	Bay leaves
pergumidiene	PPARA	Black pepper
phaeophytin A	NR5A2; PPARA	Dill
phaeophytin B	AMY2A; PPARA	Dill
phaseollinisoflavan	AMY2A; DPP4; HSD11B1; NR5A2; PDK2; PPARA	Liquorice
phenylacetic acid	MGAM	Black pepper; saffron
Phenylpropanoid glucoside derivative	FBP1; MGAM; PPARA	Star anise
phlorizin	AMY2A; MGAM; NR5A2; PDK2	Fenugreek
p-hydroxybenzoic acid-O-glucoside	RBP4	Fennel
phytadiene	RBP4	Bay leaves
picein	RBP4	Sage
pimentol	DPP4; MGAM; PPARA	Allspice
pinocembrin	MGAM; NR5A2; RBP4	Liquorice
piperamine	MGAM; NR5A2; RBP4	Black pepper
piperanine	AKR1B1; MGAM; NR5A2; PPARA; RBP4	Black pepper
piperchabamide	RBP4	Black pepper
pipercyclobutanamide A	AKR1B1; AMY2A; DPP4; FBP1; GCK; INSR; MGAM; NR5A2; PDK2; PPARA; PPARD; PPARG; PTPN9; PYGL	Black pepper
pipercyclobutanamide B	AKR1B1; AMY2A; DPP4; FBP1; GCK; INSR; MGAM; NR5A2;	Black pepper

	PDK2; PPARA; PPARD; PPARG; PTPN9; PYGL	
piperettine	MGAM; NR5A2; PPARA	Black pepper
pipericide	PPARA	Black pepper
piperine	MGAM; RBP4	Black pepper; ginger
piperolein A	AKR1B1; PPARA; RBP4; RXRA	Black pepper
piperolein B	AKR1B1; MGAM; NR5A2; PPARA; RBP4; RXRA	Black pepper
piperylin	MGAM; RBP4	Black pepper
p-menthane-2,6,8,9-tetrol-9-glucoside	FBP1; MGAM	Caraway
postlupulone	MGAM; NR5A2	Hops
prehumulone	MGAM; NR5A2	Hops
prelupulone	MGAM; NR5A2	Hops
prenyllicoflavone A	AMY2A; DPP4; FBP1; HSD11B1; MGAM; NR5A2; PDK2; PPARA; PPARG	Liquorice
procurcumenol	RBP4	Turmeric
procyanidin B2	AMY2A; HSD11B1; MGAM; PDK2; PTPN9	Cinnamon
prunetin	AMY2A; PPARA; RBP4	Liquorice
psilostachyin C	NR5A2; PTPN9; RBP4	Yarrow
psoralen	RBP4	Parsley
pyrethrin I	MGAM; RBP4	Yarrow
quercetin	AMY2A; MGAM; NR5A2; PPARA; RBP4	Allspice; aniseed; black pepper; cinnamon; clove; cumin; dill; fennel; fenugreek; hops; lemon balm;

		lemongrass; marjoram; oregano; parsley; rosemary; saffron; sage; star anise; thyme; yarrow
quercetin 3-(2Gal-rhamnosyl-robinobioside)	AMY2A; DPP4; FBP1; MGAM; PDK2; PPARA; PTPN9	Turmeric
quercetin 3-rhamnosyl-(1 -2)-rhamnoside	AMY2A; DPP4; FBP1; HSD11B1; MGAM; NR5A2; PDK2; PTPN9; PYGL	Turmeric
quercetin-3-O-(2-O-galloyl)-B-D-glucoside	AMY2A; DPP4; MGAM; PDK2; PTPN9; PYGL	Allspice
quercetin-3-O-a-L-arabinoside (avicularin)	MGAM; PDK2	Allspice
quercetin-3-O-B-D-glucuronide-6"-methyl-ester	MGAM; NR5A2; PTPN9	Allspice
quercetin-3-O-caffeoylglucoside	AKR1B1; AMY2A; DPP4; FBP1; HSD11B1; INSR; MGAM; NR5A2; PDK2; PPARA; PPARD; PPARG; PTPN9; PYGL	Caraway
quercetin-3-O-galactoside (hyperoside)	AMY2A; MGAM	Allspice; black pepper; fennel; star anise
quercetin-3-O-glucoside (isoquercitrin)	AMY2A; MGAM; NR5A2	Bay leaves; black pepper; caraway; cumin; dill; fennel; lemon balm; liquorice; oregano; star anise; thyme
quercetin-3-O-rhamnoside	AMY2A; INSR; MGAM; NR5A2	Bay leaves; black pepper; star anise; turmeric
quercetin-3-O-xyloside	AMY2A; DPP4; FBP1; MGAM; PDK2;	Star anise

	PPARA; PPARG; PTPN9; PYGL	
quercetin-7-O-glucoside	AMY2A; FBP1; MGAM; PPARA; PPARG; PTPN9	Oregano; sage; thyme
quercetin-7-O-methyl	AKR1B1; AMY2A; DPP4; FBP1; HSD11B1; MGAM; NR5A2; PPARA; PTPN9; RBP4	Oregano
quercetin-O-glucuronide (miquelianin)	AMY2A; DPP4; FBP1; HSD11B1; MGAM; NR5A2; PDK2; PPARA; PTPN9; PYGL	Allspice; aniseed; caraway; dill; fennel
quercitrin	AMY2A; INSR; MGAM; NR5A2; PTPN9	Hops; lemongrass
rel-(5S,6S,7R,10R,12S,13R)-7-hydroxyapiana-8,14-diene-11,16-dion-(22,6)-olide	MGAM; NR5A2	sage
rel-(5S,6S,7S,10R,12S,13R)-7-hydroxyapiana-8,14-diene-11,16-dion-(22,6)-olide	AMY2A; NR5A2	sage
retrofractamide A	AKR1B1; NR5A2; PPARA; RBP4	Black pepper
rhamnetin	AMY2A; MGAM; RBP4	allspice; clove
rhamnetin-O-triglucoside	AMY2A; DPP4	Black pepper
rhamnocitrin	AMY2A; RBP4	Clove; lemon balm
rosmanol	MGAM	Rosemary; sage
rosmaridiphenol	MGAM; NR5A2	Rosemary
rosmarinic acid	AKR1B1; MGAM; PPARA; PTPN9; RBP4	Aniseed; basil; black pepper; caraway; cinnamon; cumin; dill; fennel; lemon balm; lemongrass; marjoram; oregano; rosemary; sage; thyme; yarrow

rosmarinic-acid-3-O-glucoside	DPP4; HSD11B1; MGAM; PPARA; PTPN9	Rosemary; thyme
rutin	AMY2A; DPP4; FBP1; HSD11B1; MGAM; NR5A2; PDK2; PTPN9; PYGL	Aniseed; basil; black pepper; cinnamon; cumin; dill; fennel; fenugreek; hops; lemon balm; marjoram; oregano; rosemary; sage; star anise; thyme; turmeric; yarrow
sagecoumarin	AMY2A; DPP4; FBP1; FFAR1; HSD11B1; MGAM; NR5A2; PDK2; PPARA; PPARD; PPARG	Sage
sageone	MGAM; RBP4	Sage
sagequinone methide A	MGAM	Sage
sagerinic acid	AKR1B1; AMY2A; DPP4; FBP1; HSD11B1; MGAM; NR5A2; PDK2; PPARA; PPARD; PTPN9; PYGL	Lemon balm; sage
sakuranetin	PPARA	Thyme
salofficinoid E	MGAM; PPARA; RBP4	Sage
salofficinoid G	NR5A2	Sage
salofficinoid H	RBP4	Sage
salvianolic acid A	AKR1B1; AMY2A; MGAM; NR5A2; PDK2; PPARA; PPARG	Lemon balm
salvianolic acid B	AMY2A; DPP4; FBP1; HSD11B1; MGAM; PDK2; PPARA;	Lemon balm; sage

	PPARD; PPARG; PTPN9; PYGL	
salvianolic acid C	AMY2A; DPP4; FBP1; HSD11B1; MGAM; NR5A2; PDK2; PPARA; PPARD; PPARG; PYGL	Lemon balm
salvianolic acid F	AMY2A; MGAM; RBP4	Lemon balm
salvianolic acid K	AMY2A; DPP4; FBP1; MGAM; PPARA; PPARG; PYGL	Thyme
salvianolic acid L	AMY2A; DPP4; MGAM; NR5A2; PPARA; PPARD; PTPN9	sage
santamarin	NR5A2; RBP4	Bay leaves
saponarin	AMY2A; MGAM; NR5A2	Sage
sarcaglaboside D	AMY2A; HSD11B1; NR5A2; PTPN9	Liquorice
sarsasapogenin	AMY2A; DPP4; HSD11B1; NR5A2	Fenugreek
saussurea lactone	RBP4	Yarrow
saxalin	RBP4	Parsley
schaftoside	AMY2A; DPP4; FBP1; MGAM	Liquorice; yarrow
scutellarein	AMY2A; MGAM; NR5A2; PTPN9; RBP4	Rosemary
secotanapartholide A	RBP4	Yarrow
shinflavanone	AKR1B1; AMY2A; DPP4; FBP1; GCK; HSD11B1; INSR;	Liquorice

	MGAM; NR5A2; PDK2; PPARA; PPARD; PPARG; PTPN9; PYGL	
shinpterocarpin	AKR1B1; AMY2A; HSD11B1; MGAM; NR5A2; PDK2; PTPN9; RBP4; RXRA	Liquorice
shogaol	RBP4	Ginger; rosemary
sideritoflavone	RBP4	Thyme
sintenin	NR5A2	Yarrow
smilagenin	AMY2A; DPP4; HSD11B1; NR5A2	Fenugreek
sophoraflavonoloside	AMY2A; FBP1; MGAM; NR5A2; PTPN9	Saffron
spathulenol	RBP4	Allspice; aniseed; basil; black pepper; bay leaves; caraway; cardamom; dill; liquorice; marjoram; oregano; thyme; yarrow
stigmasat-3,5-dien-7-one	AMY2A; MGAM; NR5A2	Thyme
stigmast-4-en-3-one	AMY2A; DPP4; MGAM; NR5A2; PPARD	Bay leaves
swertiajaponin	FBP1; MGAM	Lemongrass
syringin	PPARA	Caraway; fennel
syringin-4-O-b-glucoside	PPARA	Fennel
syzyginin A	AMY2A; DPP4; MGAM; PDK2	Clove

syzyginin B	AMY2A; DPP4; FBP1; MGAM; PDK2; PPARA; PTPN9	Clove
tamarixetin-3-O-B-D-glucopyranoside	AMY2A; MGAM; PTPN9	Clove
tashironin	AMY2A; DPP4; NR5A2; PTPN9	Star anise
tashironin A	AMY2A; NR5A2	Star anise
taxifolin	AKR1B1; AMY2A; DPP4; GCK; HSD11B1; MGAM; NR5A2; PDK2; PPARA; PTPN9; PYGL	Oregano; thyme
taxifolin-7-O-hexoside (dihydroquercetin-7-glucoside)	FBP1; MGAM; PPARA; PTPN9	Saffron
t-cadinol	RBP4	Allspice; cinnamon; lemongrass; oregano
tectochrysin	MGAM; RBP4	Rosemary
tetradeca-2E,4E,12Z-triene-8,10-diynoic acid isobutylamide	RBP4	Yarrow
tetrahydroxycurcumin	AMY2A; MGAM; PDK2; PPARA; PTPN9	Turmeric
tetrahydroxymethoxychalcone	MGAM; RBP4	Liquorice
thymol	MGAM	Basil; bay leaves; caraway; cardamom; clove; cumin; dill; fennel; lemon balm; liquorice; marjoram; oregano; rosemary; thyme; turmeric; yarrow
thymonin	RBP4	Thyme
thymusin	RBP4	Thyme
tigogenin	AMY2A; DPP4; HSD11B1; MGAM; NR5A2	Fenugreek

torreyol	RBP4	Allspice; basil; black pepper; lemongrass; thyme; yarrow
trachyone	AKR1B1; NR5A2	Black pepper
trans_miyabenol_C	AMY2A; MGAM; PPARA; PTPN9; PYGL	Fennel
trans_Muurolol	RBP4	Allspice; black pepper; cardamom
trans_resveratrol_3_O_b_D_glucopyranoside	AMY2A; MGAM; PPARA; PTPN9	Fennel
trans-a-bergamotene	RBP4	Basil; black pepper; cumin; dill; ginger; hops; lemongrass; marjoram; nutmeg; oregano; star anise; thyme; turmeric
trans-crocetin-(B-D-neopolitanosyl)-(B-D-gentiobiosyl)-ester (trans-5-nG)	AMY2A; FBP1; MGAM	Saffron
trans-crocetin-(B-D-neopolitanosyl)-(B-D-glucosyl)-ester (trans-4-nG)	AMY2A	Saffron
trans-crocetin-(tri-B-D-glucosyl)-(B-D-gentiobiosyl)-ester (trans-5-tG)	AMY2A; DPP4; FBP1; MGAM; PDK2	Saffron
tribulesterine	AMY2A; RBP4	Saffron
tricholein	AKR1B1; RBP4	Black pepper
trichostachine	MGAM; RBP4	Black pepper
tricin-7-O-D-glucopyranoside	AMY2A; FBP1; MGAM; PPARA	Fenugreek
trigofoenoside A	AMY2A; FBP1; MGAM; NR5A2; PPARA; PTPN9	Fenugreek
trigofoenoside B	AMY2A; FBP1; MGAM; PDK2; PTPN9	Fenugreek
trigofoenoside C	AMY2A; DPP4; FBP1; MGAM	Fenugreek
trigofoenoside D	AMY2A; DPP4; FBP1; MGAM; DPK2; PTPN9	Fenugreek

trigofoenoside F	AMY2A; DPP4; MGMAM; NR5A2; PDK2; PTPN9; PYGL	Fenugreek
trigofoenoside G	AMY2A; DPP4; NR5A2; PDK2; RBP4	Fenugreek
trigoneoside Xa	AMY2A; DPP4; MGAM; NR5A2; PDK2; PTPN9	Fenugreek
trigoneoside Xb	AMY2A; DPP4; MGAM; NR5A2; PDK2; PTPN9	Fenugreek
trigoneoside XIb	AMY2A; DPP4; MGAM; PTPN9	Fenugreek
trigoneoside XIIa	AMY2A; DPP4; NR5A2	Fenugreek
trigoneoside XIIb	AMY2A; DPP4; NR5A2	Fenugreek
trigoneoside XIIIa	AMY2A; DPP4; MGAM	Fenugreek
turmeronol A	RBP4	Turmeric
turmeronol B	RBP4	Turmeric
tyrosine	MGAM	Fenugreek
umbelliprenin	AKR1B1; NR5A2; PPARA; RBP4; RXRA	Aniseed; dill
uralsaponin C	AMY2A; DPP4; FBP1; MGAM; PDK2; PTPN9; PYGL	Liquorice
ursolic acid	AMY2A	Basil; lemon balm; rosemary; sage
valencene	RBP4	Basil; bay leaves; hops; lemongrass; sage
verimol H	AMY2A	Star anise
verimol K	RBP4	Star anise
vetivene	RBP4	Dill

vicenin 1	AMY2A; DPP4; FBP1; INSR; MGAM; NR5A2; PYGL	Fenugreek
vicenin 2	DPP4; FBP1; MGAM; NR5A2; PTPN9	Liquorice; marjoram; sage; thyme; yarrow
viridifloral	RBP4	Allspice; basil; lemongrass; oregano; sage; thyme
vitamin B2	MGAM; RBP4	Fenugreek; saffron
vitamin E	PPARA	Bay leaves; cardamom; ginger; paprika; saffron; thyme
vitexin	AMY2A; FBP1; MGAM; NR5A2; PPARA; PTPN9	Fenugreek; yarrow
wogonoside	AMY2A; MGAM; PPARA	Oregano
xambioona	AKR1B1; AMY2A; DPP4; FBP1; GCK; HSD11B1; INSR; MGAM; NR5A2; PDK2; PPARA; PPARD; PPARG; PTPN9; PYGL	Liquorice
xanthohumol	AKR1B1; AMY2A; MGAM; PPARA; RBP4	Hops
xanthohumol B	MGAM; RXRA	Hops
xanthohumol C	AKR1B1; AMY2A; MGAM; NR5A2; PPARA	Hops
xanthohumol D	AKR1B1; MGAM; PPARA; PTPN9	Hops
xanthohumol G	MGAM; PTPN9	Hops

xanthohumol I	AKR1B1; MGAM; NR5A2; PPARA; RXRA	Hops
xanthohumol M	MGAM	Hops
xanthomicrol	RBP4	Thyme
xanthorrhizol	RBP4	Turmeric
xanthotoxin	RBP4	Parsley
xanthuhumol derivative 7	MGAM; PPARA; PTPN9	Hops
yamogenin	AMY2A; DPP4; HSD11B1; MGAM; NR5A2	Fenugreek
yuccagenin	AMY2A; DPP4; HSD11B1; NR5A2	Fenugreek
yunganoside A1	AMY2A; DPP4; FBP1; MGAM; PDK2; PTPN9; PYGL	Liquorice
yunganoside B1	AMY2A; DPP4; MGAM; PDK2; PTPN9	Liquorice
yunganoside J1	AMY2A; DPP4; FBP1; MGAM; NR5A2; PDK2; PTPN9	Liquorice
yunganoside K2	AMY2A; DPP4; FBP1; MGAM; NR5A2; PDK2; PTPN9; PYGL	Liquorice
yunganoside L1	AMY2A; DPP4; FBP1; MGAM; PDK2; PTPN9; PYGL	Liquorice
yunganoside L2	AMY2A; DPP4; FBP1; MGAM; NR5A2; PDK2; PTPN9; PYGL	Liquorice

yunnaneic acid F	AMY2A; HSD11B1; MGAM; NR5A2; PPARA; PTPN9; PYGL	Lemon balm
yunnanglysaponin B	AMY2A; DPP4; FBP1; INSR; MGAM; PDK2; PTPN9; PYGL	Liquorice
zaluzanin D	NR5A2	Bay leaves
zingiberene	RBP4	Aniseed; caraway; ginger; lemongrass; turmeric
zizybeoside I	MGAM; PTPN9	Fennel
